# Multiple myeloma immunoglobulin lambda translocations portend poor prognosis

**DOI:** 10.1038/s41467-019-09555-6

**Published:** 2019-04-23

**Authors:** Benjamin G. Barwick, Paola Neri, Nizar J. Bahlis, Ajay K. Nooka, Madhav V. Dhodapkar, David L. Jaye, Craig C. Hofmeister, Jonathan L. Kaufman, Vikas A. Gupta, Daniel Auclair, Jonathan J. Keats, Sagar Lonial, Paula M. Vertino, Lawrence H. Boise

**Affiliations:** 1 Department of Hematology and Medical Oncology, Emory University School of Medicine, 1365 Clifton Rd. NE, Atlanta, GA 30322 USA; 2 Department of Radiation Oncology, Emory University School of Medicine, 1701 Uppergate Drive, Atlanta, GA 30322 USA; 3 Winship Cancer Institute, Emory University, 1365 Clifton Rd, Atlanta, GA 30322 USA; 4 Charbonneau Cancer Research Institute, University of Calgary, 3330 Hospital Drive, Calgary, AB T2N 4N1 Canada; 5 Department of Pathology and Laboratory Medicine, Emory University School of Medicine, Atlanta, GA 30322 USA; 6 Multiple Myeloma Research Foundation, 383 Main Avenue, 5th Floor, Norwalk, CT 06851 USA; 7 Translational Genomics Research Institute, 445 North Fifth Street, Phoenix, AZ 85004 USA; 8 Present Address: Department of Biomedical Genetics, University of Rochester School of Medicine and Dentistry, Rochester, NY 14642 USA

**Keywords:** Cancer genomics, Myeloma, Tumour biomarkers

## Abstract

Multiple myeloma is a malignancy of antibody-secreting plasma cells. Most patients benefit from current therapies, however, 20% of patients relapse or die within two years and are deemed high risk. Here we analyze structural variants from 795 newly-diagnosed patients as part of the CoMMpass study. We report translocations involving the immunoglobulin lambda (IgL) locus are present in 10% of patients, and indicative of poor prognosis. This is particularly true for IgL-MYC translocations, which coincide with focal amplifications of enhancers at both loci. Importantly, 78% of IgL-MYC translocations co-occur with hyperdiploid disease, a marker of standard risk, suggesting that IgL-MYC-translocated myeloma is being misclassified. Patients with IgL-translocations fail to benefit from IMiDs, which target IKZF1, a transcription factor that binds the IgL enhancer at some of the highest levels in the myeloma epigenome. These data implicate IgL translocation as a driver of poor prognosis which may be due to IMiD resistance.

## Abstract (web summary)

Multiple myeloma is frequently characterised by translocation of genes next to the immunoglobulin heavy chain locus. In this study, the authors sequence a large cohort of high risk myeloma samples and find translocations of cMyc to the immunoglobulin heavy chain locus and this is associated with poor prognosis.

## Introduction

Multiple myeloma is the second most common hematological cancer, which affects terminally differentiated antibody secreting B cells, known as plasma cells. Clinical manifestations of myeloma include hypercalcemia, anemia, renal failure, and lytic bone lesions. There have been significant improvements in survival over the past decade, due to new therapies, which include autologous stem cell transplant^1^, proteasome inhibitors^2^, the immunomodulatory imide drugs (IMiDs), thalidomide^3^, lenalidomide^4,5^, and pomalidomide, and more recently monoclonal antibodies^6,7^. Despite these advances, ~20% of patients relapse or die within two years of diagnosis^8,9^. These patients are referred to as high risk, but many do not have known high risk features at diagnosis^10^. Understanding how to identify and treat these patients as well as the mechanisms underlying the biology of high-risk myeloma is critical for improving outcomes.

Genetic analyses of myeloma over the last quarter century have revealed a bifurcation of founding genetic alterations with approximately half of myelomas containing an immunoglobulin heavy chain (IgH) translocation^11^, which most commonly juxtapose the IgH enhancer with *CCND1* [t(11;14)], *WHSC1* [t(4;14); also known as *MMSET* and *NSD2*], or *MAF* [t(14;16)]. The other half of myeloma are hyperdiploid, which is an aneuploidy of chromosomes 3, 5, 7, 9, 11, 15, 19, and 21^12^. Despite these seemingly simple explanations of the initiating events, the manifestation of myeloma at presentation is often confounded by a complex array of genetic alterations including amplification of chromosome 1q [amp(1q)], deletion of chromosome 13 [del(13)], deletion of chromosome 17p [del(17p)], dysregulation of MYC^13^ and Cyclin D proteins^14^, as well as mutations in common and disease-specific oncogenes (KRAS, NRAS, FAM46C, DIS3, BRAF, TRAF3, TP53)^15^. Compounding the wide array of genetic abnormalities in myeloma is an extensive clonal heterogeneity, wherein selective pressures in the microenvironment and/or treatment promote the outgrowth of sub-clones harboring specific mutations that confer a survival advantage^16^. Fortunately, modern combination therapies are mostly effective despite disease heterogeneity, with the majority of patients responding to frontline treatments that target plasma cell biology rather than specific genetic lesions^17^.

To better understand the genetic basis of myeloma, and specifically high-risk disease, we investigated the genomic landscape of 795 newly diagnosed myeloma patients using long-insert whole-genome sequencing as part of the Clinical Outcomes in Multiple Myeloma to Personal Assessment (CoMMpass) study. These data identified recurrent translocations in 66.4% of newly diagnosed myeloma patients, with t(11;14), t(4;14), t(MYC), and other immunoglobulin translocations being the most common. While these translocations resulted in aberrant oncogene expression, very few were prognostic of outcome. The notable exception were patients with a translocation involving the immunoglobulin lambda (IgL) light chain locus, who experienced a significantly worse progression-free (PFS) and overall survival (OS), which was most pronounced for IgL-MYC translocations. In contrast to IgH translocations, the majority of IgL-translocations [t(IgL)] were sub-clonal and coincided with hyperdiploidy, typically considered a marker of standard risk, and thus could result in the misclassification of patients with t(IgL) myeloma. Most cases with IgL-translocations (68%) were accompanied by focal amplifications of the IgL 3’ enhancer, which is one of the most active enhancers in myeloma cells, suggesting it can robustly drive expression from transposed genes. Patients with an IgL-translocation did not benefit from IMiD-containing therapies that target the lymphocyte-specific transcription factor Ikaros (IKZF1)^18,19^, which is bound at high levels to the IgL enhancer. These data identify IgL-MYC translocations as a marker of poor prognosis, independent of other genetic abnormalities, with implications for diagnosis and treatment.

## Results

### The translocation architecture of multiple myeloma

**Fig. 1:**
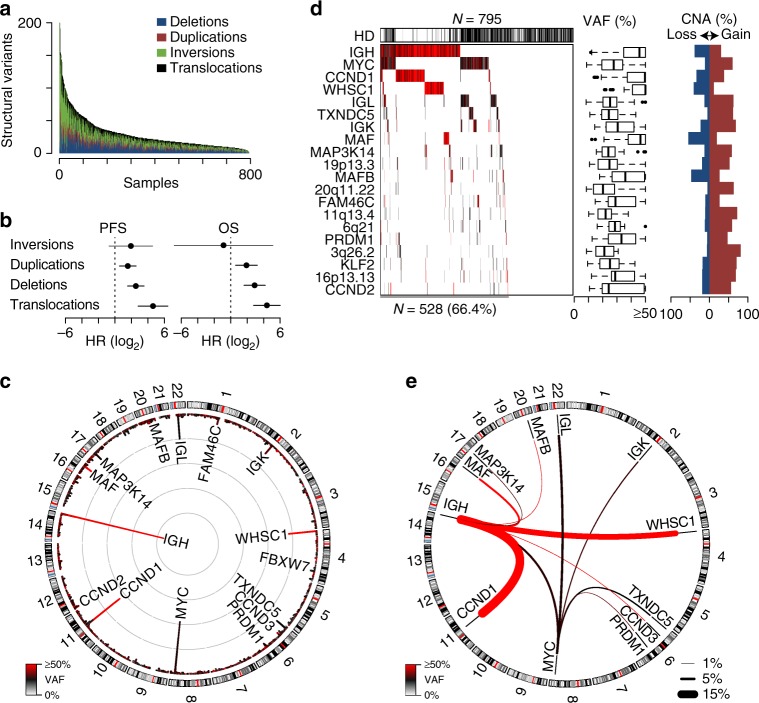
Structural variants correspond with poor prognosis. **a** The number of somatic structural variants per sample in 795 newly diagnosed myeloma specimens ordered by the total number of structural variants. Three samples are cropped at 200 structural variants, but have 1823, 529, and 257 SV. **b** Progression-free (PFS) and overall survival (OS) hazard ratios (HR) for structural variants indicating the increased hazard of having the maximum number of each structural variant, with 95% confidence intervals shown. **c** Circos plot of translocation frequency. The frequency of translocations is plotted on the inside with gray concentric circles denoting 10 percentiles per megabase and the color of each region represents the median variant allele frequency (VAF; key bottom left). Chromosome ideograms are represented and labelled on the outside. **d** Waterfall plot of translocated regions showing those present in ≥2% of newly diagnosed patients (left). Hyperdiploid (HD) status is denoted above. The VAF of each translocation is denoted in color and summarized in boxplots (middle) showing the median and quartiles with the whiskers extending to the most extreme data point within 1.5 times the interquartile range. The frequency of proximal (≤10 kb) copy number alterations (CNA) associated with each translocation are shown (right). **e** Circos plot of translocated regions prevalent in ≥1% of samples. The thickness and color of lines connecting two regions denotes the frequency and median VAF of the translocation, respectively (see keys bottom right and left). Hazard ratios were determined using a Cox proportional hazards regression Wald’s test as a function of the number of structural events. Source data are provided as a Source Data file

A comprehensive analysis of structural variants in multiple myeloma was conducted using long-insert whole-genome paired-end sequencing performed on DNA isolated from CD138+ myeloma cells and normal peripheral blood to determine cancer-specific somatic alterations as part of the CoMMpass study (NCT01454297). CoMMpass is a longitudinal study of over 1000 newly diagnosed myeloma patients enrolled from North American and European collection sites. In total, samples from 795 newly diagnosed patients were subjected to long-insert sequencing yielding an average of 304 million paired-end reads per specimen with an average fragment size of 846 bp, thus spanning the genome with 40.9× coverage. Identification of structural variants including deletions, duplications, inversions, and translocations revealed a median of 21 structural variants in newly diagnosed myeloma (Fig. 1a). Deletions, duplications, and translocations corresponded with worse PFS and OS, with translocations being the most significant (Fig. 1b).

Translocations were determined by discordant paired-end reads or by sequencing across the translocations breakpoint and were thus resolved to within 1 kb (Supplementary Data 1). Commonly translocated regions were identified using a 1 Mb window incremented by 0.5 Mb across the genome. Frequent translocations included regions proximal to *IgH* (41%), *MYC* (23%), *CCND1* (17%), *WHSC1* (11%), *IgL* (9.8%), *TXNDC5* (4.9%), *IgK* (4.3%), and *MAF* (4.1%) (Fig. 1c). In total, 20 regions were translocated at a frequency of 2% or more with 66% of newly diagnosed patients having at least one of these rearrangements (Fig. 1d, left). Of the patients with these common translocations, 42% also exhibited hyperdiploidy (HD), which was mostly confined to non-IgH translocated myeloma (Fig. 1d, top). To estimate clonality, the variant allele frequency (VAF) of each translocation was calculated such that 50% would indicate cellular clonality of a heterozygous translocation in diploid regions of the genome. This analysis identified a median VAF of 48% for IgH translocations, whereas MYC and other non-IgH translocations had median VAF of 26% and 28%, respectively (Fig. 1d, middle). Identification of recurrently translocated partner loci confirmed IgH-to-CCND1 (16%), WHSC1 (11%), MAF (3.3%), CCND3 (1.1%), and MAFB (1.0%) were clonal whereas MYC translocations, including those to all three immunoglobulin loci, were sub-clonal (Fig. 1e). Most translocations were frequently accompanied by proximal (≤10 kb) focal amplifications, but these were less common at loci with clonal IgH translocations (Fig. 1d, right, e.g., *WHSC1*, *MAF*, and *MAFB*). These data indicate that IgH translocations are mostly clonal whereas non-IgH translocations occur at sub-clonal frequencies, which is consistent with previous reports indicating IgH translocations are primary events whereas MYC translocations represent complex secondary events, and are often accompanied by duplications immediately adjacent to the translocation breakpoint^20,21^.

### IgH translocation breakpoints

**Fig. 2:**
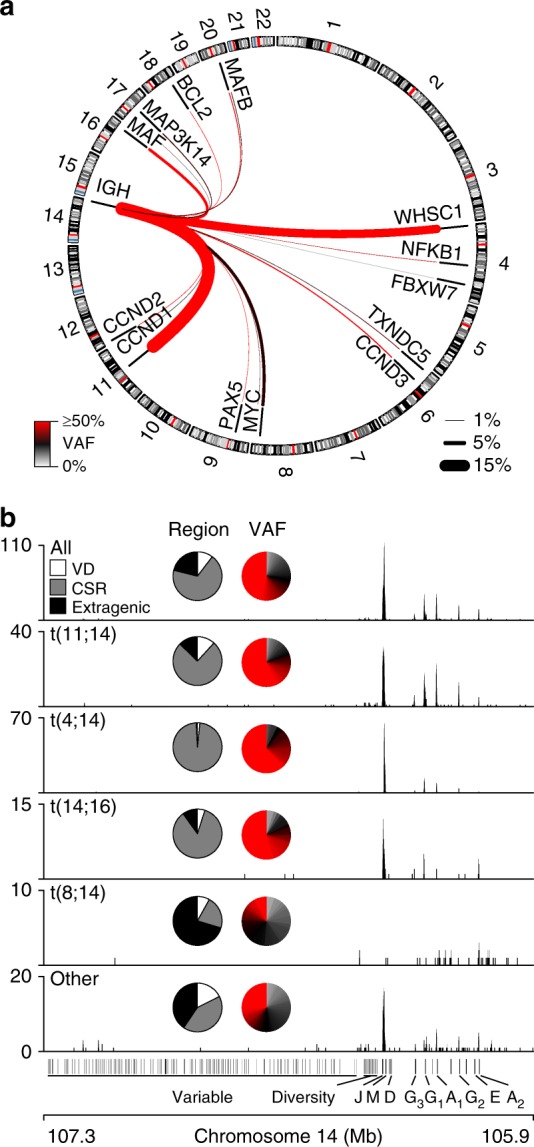
IgH translocations have distinct etiologies. **a** Circos plot of IgH translocations in newly diagnosed myeloma patients (*N* = 795). The frequency and median VAF of each type of translocation is denoted by the thickness and color of the line, respectively (key bottom left and right). **b** Genome (GRCh37) plots of translocations across the IgH region for all IgH translocations (top) or specific types of IgH translocations (below). Inset are pie charts depicting the percentage of translocations that occur in the variable and diversity (VD), class switch recombination (CSR) (+/−2.5 kb), and extragenic regions (left) and the VAF of each translocation. Source data are provided as a Source Data file

The significance of translocations was studied starting with the most frequent region, the IgH locus. The most common IgH translocations included t(11;14), t(4;14), t(8;14), and t(14;16), which accounted for 83% of all IgH-translocated patients (Fig. 2a). These translocations resulted in aberrant upregulation of the transposed gene (Supplementary Fig. 1a). IgH translocations preferentially occurred near the class switch recombination (CSR) regions located at the 5’ edge of the constant heavy chains μ (M), γ_1_ (G_1_), α_1_ (A_1_), γ_2_ (G_2_), α_2_ (A_2_), and γ_3_ (G_3_) (Fig. 2b). However, there were distinct differences between the IgH translocation species. For instance, t(11;14), t(4;14), and t(14;16) translocations were primarily located at CSR regions and had a VAF approaching 50% indicating that these translocations were clonal, with t(11;14) translocations more likely to occur at the G_1_, A_1_, G_2_, A_2_, G_3_ CSR regions, and t(4;14) almost exclusively at the M switch region. In contrast, t(8;14) and other IgH translocations preferentially occurred at extragenic regions and had lower VAF, indicating that the majority of these alterations occur in a sub-clonal fraction of the disease (Fig. 2b). To determine if there were differences in breakpoint location between the types of t(IgH), translocations arising from the CSR, VD, and extragenic regions were quantified and biases in location were expressed as an odds ratio of overlap for each region and translocation (Supplementary Fig. 1b). These data further indicated that t(11;14), t(4;14), and t(14;16) translocations preferentially occurred at the CSR regions, with t(4;14) showing the strongest enrichment. Conversely, t(8;14) and other IgH translocations preferentially occurred at extragenic regions (Supplementary Fig. 1b, right). These data suggest that the majority of IgH translocations are clonal initiating events that occurred during germinal center B cell CSR, which is consistent with previous observations^11^. However, IgH-MYC translocations seem to be distinct from other IgH translocations in that they are sub-clonal and have breakpoints distal to CSR regions suggesting they likely occur by a different mechanism and at a later time point in myelomagenesis.

### MYC translocations are juxtaposed throughout the genome

**Fig. 3:**
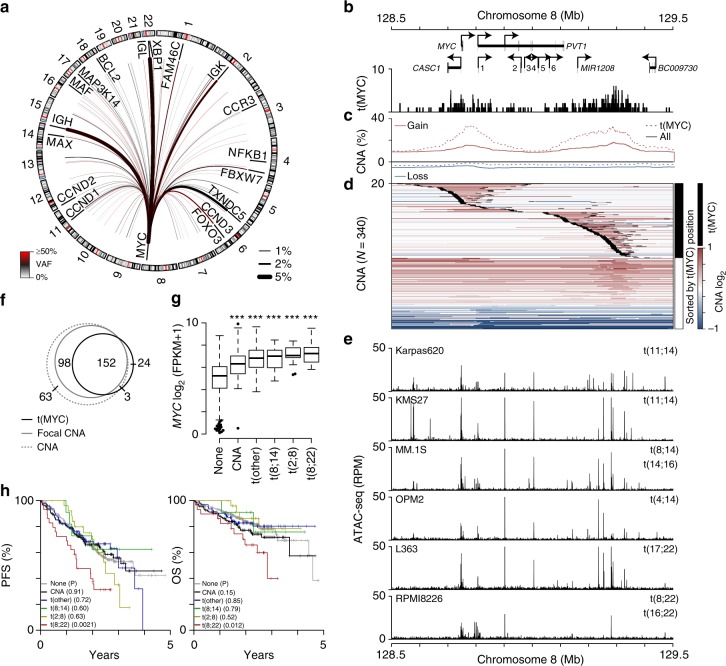
MYC translocations correspond with focal amplifications and aberrant expression. **a** Circos plot of MYC translocations in newly diagnosed myeloma (*N* = 795). The median VAF and frequency of each translocation is denoted by the color and thickness of the line, respectively (see keys bottom left and right). **b** Genome (GRCh37) plot of the MYC locus showing genes (arrows indicate the direction of transcription; 1: *MIR1204*, 2: *TMEM75*, 3: *MIR1205*, 4: *U4*, 5: *MIR1206*, 6: *MIR1207*), and the location of MYC translocations [t(MYC)]. **c** Frequency of copy number gains (red) and losses (blue) for all samples (solid line) and those with t(MYC) (dashed line). **d** A heatmap of copy number across the locus is shown for samples with either a MYC translocation (black) or only a CNA (white) sorted by the location of the MYC translocation, which are superimposed on the heatmap as 10 kb black regions. **e** ATAC-seq for 6 myeloma cell lines across the locus is shown below (RPM: reads per million). **f** Venn diagram of samples with a CNA at the MYC locus (dashed gray line), CNA boundary (solid gray line), or MYC translocation (black line). **g** Gene expression of *MYC* in samples with RNA-seq data (*N* = 611) for patients with no MYC alteration (none; *N* = 382), MYC CNA only (*N* = 83), MYC translocations to non-immunoglobulin genes [t(other); *N* = 81], IgH-MYC translocations [t(8;14); *N* = 27], IgK-MYC translocations [t(2;8); *N* = 13], and IgL-MYC translocations [t(8;22); *N* = 25]. ***P < 0.001, analysis of variance with Tukey’s post hoc test. Boxplots show the median and quartiles with the whiskers extending to the most extreme data point within 1.5 times the interquartile range. **h** Progression-free (PFS; left) and overall survival (OS; right) for patients stratified by MYC structural variant type as in part **g**. *P*-values are shown in parenthesis and were calculated using a Cox proportional hazards Wald's test and denote the significance of survival differences relative to myelomas without a detectable MYC structural variant (none). Source data are provided as a Source Data file

Since IgH-MYC translocations had a pattern of breakpoints that was distinct from most other IgH translocations, the MYC translocation landscape was examined. MYC translocations occurred in 182 (23%) of 795 patients, and were juxtaposed to a large number of regions (Fig. 3a). The most common regions included loci near *IgL* [t(8;22); *N* = 32], *IgH* [t(8;14); *N* = 30], *TXNDC5* (*N* = 19), *IgK* [t(2;8); *N* = 16], *FOXO3* (*N* = 8), and *FAM46C* (*N* = 6). MYC translocations had a median VAF of 26% with breakpoints clustered across two broad regions, one centered on *MYC* and the other 600 kb to the telomeric side of *MYC*, downstream of *PVT1* (Fig. 3b). Focal Copy number alteration (CNA) were enriched in the same regions where MYC translocation breakpoints occurred and this was particularly prevalent in MYC-translocated myeloma (Fig. 3c). Indeed, a heatmap of CNA across the *MYC* locus sorted by the coordinate of the translocation breakpoint revealed that most MYC translocations (black) demarcated the borders of focal amplifications (Fig. 3d). MYC translocation breakpoints and CNA overlapped several regions of chromatin accessibility in myeloma cell lines (Fig. 3e) that coincide with known enhancers as well as insulator elements that delineate *MYC* and *PVT1* promoter-enhancer interactions^22^. In total, 85% of MYC translocations were within 10 kb of a focal CNA boundary (Fig. 3f). Both types of genomic alterations resulted in increased *MYC* expression, but there was no difference in expression levels between those myelomas with only MYC CNA and those with MYC translocations (Fig. 3g). Interestingly, despite similar levels of baseline MYC expression, distinct MYC-alterations exhibited differences in outcome. Indeed, among MYC structural alterations, only those patients with IgL-MYC translocations had a significantly worse PFS and OS as compared to those patients with no MYC alteration (Fig. 3h). These data indicate that aberrant MYC expression resulting from MYC amplification or translocation is a common feature of myeloma, but the IgL-MYC translocated subset is unique among MYC alterations in that it portends a poor prognosis.

### IgL-MYC translocations are a marker of poor prognosis

**Fig. 4:**
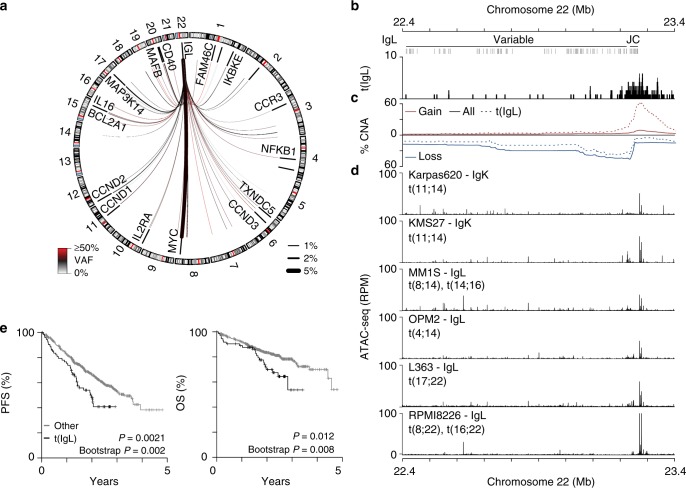
IgL translocations portend poor prognosis. **a** Circos plot showing the repertoire of IgL translocations in newly diagnosed myeloma where line color and thickness denote variant allele frequency (VAF) and translocation frequency (keys bottom left and right). **b** Genome plot (GRCh37) of the IgL locus showing the variable, joining (J), and constant (C) regions and locations of translocation breakpoints. **c** The frequency of copy number gains (red) and losses (blue) in the entire cohort (solid line) and in t(IgL) samples (dashed line), and **d** ATAC-seq for 6 myeloma cell lines labelled with their light chain expression status and known translocations denoted. **e** Kaplan-Meier analysis of IgL translocated [t(IgL)] patients (*N* = 78) as compared to non-t(IgL) (*N* = 717) for progression-free (PFS; left) and overall survival (OS; right). *P*-values were calculated using a Cox proportional hazards Wald’s test or Bootstrapped based P-value with 1000 permutations based on the hazard ratio. Source data are provided as a Source Data file

The above findings prompted us to examine IgL translocations in more detail. IgL was translocated in 9.8% (*N* = 78/795) of newly diagnosed myeloma with 41% of IgL translocations being juxtaposed to MYC and the remaining occurring throughout the genome, which included translocations proximal to *MAP3K14*, *CD40*, *MAFB*, *TXNDC5*, *CCND1*, *CCND2*, and *CCND3* albeit at much lower frequencies (7.6–1.3%; Fig. 4a). IgL translocations were primarily clustered at the 3′ end of IgL near the joining and constant (JC) regions (Fig. 4b). Similar to MYC, IgL-translocations were accompanied by focal amplifications, which were found in 68% of cases and centered at 3′ end of IgL (Fig. 4c). In fact, the most common amplified region was centered directly above the IgL 3′ enhancer, which displayed constitutive chromatin accessibility in IgK-expressing (Karpas620, KMS27), IgL-expressing (MM.1S, OPM2), and IgL-translocated (L363, RPMI8226) myeloma cell lines (Fig. 4d). As suggested by the MYC analysis, t(IgL) patients experienced a worse PFS and OS as compared to non-t(IgL) myeloma (Fig. 4e). The correspondence of t(IgL) and poor prognosis was further confirmed by bootstrap analysis, and was not attributable to differences in age, disease stage, sex, race, M-protein, or β2-Microglobulin levels (Supplementary Fig. 2a–f). Furthermore, t(IgL) patients were treated with similar front-line therapy components as compared to other patients (Supplementary Fig. 2g). Multivariate survival analysis was performed to identify any potentially confounding factors, and t(IgL) remained a significant marker of poor outcome when considering variables prognostic in univariate analysis including age, gender, and disease stage (Supplementary Fig. 2h). Additionally, patients with t(IgL) had a worse outcome than those with t(IgH) or t(IgK) (Supplementary Fig. 3a).

Survival of different subsets of t(IgL) myeloma patients were compared in the context of juxtaposed loci and IgL structural variants. First, comparison of patients with IgL-MYC (*N* = 32) and IgL-non-MYC (*N* = 46) translocations showed that patients with IgL-MYC translocations had a reduced average survival as compared to patients with no IgL translocation (Supplementary Fig. 3b). Similarly, t(IgL) patients with IgL amplification (*N* = 51) also had reduced survival compared to non-t(IgL) patients (Supplementary Fig. 3c). These two subsets largely overlapped, with 81% of patients with IgL-MYC translocations also containing an IgL amplification (Supplementary Fig. 3d). These data suggest that the specific structural variant architecture and juxtaposed oncogene influence disease aggressiveness.

To determine whether there might be other molecular features that contribute to the poor outcomes of t(IgL) patients, the mutational repertoire of t(IgL) myeloma was interrogated using high-depth exome sequencing on 783 specimens. This analysis indicated that there was no difference in the total and nonsynonymous mutational burden or mutational spectrum between t(IgL), t(IgK), t(IgH), and other myelomas (Supplementary Fig. 4a–c). Analysis of specific mutations indicated that IgL-translocated myeloma contained a similar frequency of mutations in commonly mutated genes with no statistical differences as compared to non-IgL-translocated myelomas (Supplementary Fig. 4d). A bivariate analysis considering each mutation in combination with t(IgL), identified t(IgL) as prognostic of poor outcome independent of any other common mutation (Supplementary Fig. 4e, right).

### IgL-translocated myeloma is not defined by gene expression

**Fig. 5:**
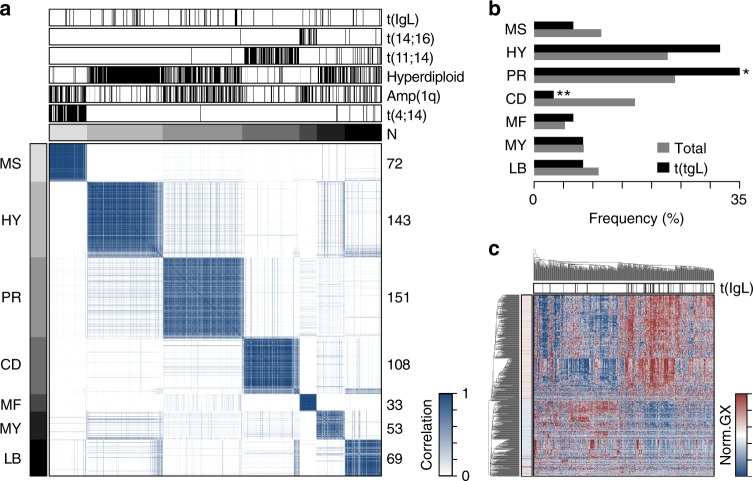
IgL translocations occur in all gene expression subtypes. **a** Consensus clustering of 629 newly diagnosed myelomas with whole genome sequencing and RNA-seq. Expression subtypes are denoted by gray annotation bars (top and left), and labels correspond with those defined by Zhan et al.^24^. The number of samples are denoted on the right. **b** Frequency of gene expression subtypes in all myelomas (gray) and in t(IgL) myeloma (black). **c** Heatmap of genes differentially expressed in t(IgL) myeloma as compared to others. Samples (columns) are clustered with t(IgL) status annotated (top; black) and gene significance denoted (left). *P < 0.05; **P < 0.01, two-sided Fisher’s exact test. Source data are provided as a Source Data file

To gain insight into the pathogenesis of IgL translocation, the relationship between t(IgL) and gene expression subtypes was determined using consensus clustering^23^ on 629 samples for which whole genome sequencing and RNA-seq data were available. Similar to previous reports^24^, this analysis identified 7 gene expression subtypes, where samples within a given subtype were highly correlated with each other but not with samples from other subtypes (Fig. 5a). Differentially regulated genes in each cluster were determined (Supplementary Fig. 5a; Supplementary Data 2), and annotated using gene set enrichment analysis (GSEA; Supplementary Data 3)^25^. This matched 5 of the 7 expression subtypes identified here with the previously defined MMSET (MS), hyperdiploid (HY), proliferation (PR), Cyclin D (CD), and MAF (MF) gene expression subtypes^24^ (Supplementary Fig. 5b). Group six corresponded with genes dysregulated in a MYC and BCL2L1 driven mouse model of multiple myeloma^26^ as well as expression of more innate B cell and/or myeloid genes and is subsequently referred to as the myeloid subtype (MY). Finally, the seventh group modestly corresponded to the low bone (LB) disease signature, which was previously noted to have a non-distinct gene expression pattern^24^ (Fig. 5a; Supplementary Fig. 5b). As expected, gene expression groups corresponded with genetic abnormalities where the MS subtype harbored t(4;14) and amp(1q), the HY gene expression subtype corresponded with genetic hyperdiploidy, the PR subtype contained amp(1q), the CD subtype had t(11;14), and MF had t(14;16) (Fig. 5a; Supplementary Fig. 5c). Notably, IgL translocations were found in every expression subtype with a modest enrichment in the PR subtype and depletion in the CD subtype (Fig. 5b).

To identify putative molecular mechanisms contributing to t(IgL) pathogenesis, genes differentially expressed in t(IgL) were determined, revealing 406 upregulated and 232 downregulated genes in t(IgL) myeloma as compared to non-t(IgL) myeloma (Supplementary Data 4; FDR < 0.01). Expression of these genes modestly aggregated t(IgL) myelomas using hierarchical clustering (Fig. 5c), but clearly grouped many non-t(IgL) with t(IgL) myelomas, suggesting that t(IgL) myeloma is not clearly defined by a baseline gene expression signature. GSEA identified overexpression of ribosomal genes, oxidative phosphorylation and respiratory electron transport chain, and MYC-regulated genes, including *MYC* itself, in t(IgL) myeloma (Supplementary Fig. 6a, top; Supplementary Data 5). Genes downregulated in t(IgL) myeloma, included those normally repressed during B cell to plasma cell differentiation as well as genes involved in cytokine and chemokine signaling (Supplementary Fig. 6a, bottom). However, even the most significant genes showed only subtle differences between t(IgL) and non-t(IgL) myeloma (Supplementary Fig. 6b). These data indicate that t(IgL) occurs across all gene expression subtypes of myeloma and does not drive a unique gene expression program.

### IgL translocations co-occur with hyperdiploid disease

As t(IgL) was not associated with any specific mutations and had only modest correlations with gene expression, we investigated structural variants that might be associated with t(IgL). The frequency of specific loci translocated directly to IgL as well as those that co-occur with t(IgL) were compared to myeloma with IgH and IgK translocations (Supplementary Fig. 7a). This indicated that IgH was more frequently translocated to *CCND1*, *WHSC1*, and *MAF*, whereas IgK and IgL were more frequently translocated to *MYC*. Approximately 40% of both IgK and IgL translocations were to *MYC* and another 20% of both t(IgK) and t(IgL) myelomas contained a *MYC* translocation but to a different locus (Supplementary Fig. 7b). IgL-translocated myeloma contained very few unique translocation partners, in that most loci transposed to IgL were also transposed to IgK or IgH. The most common translocations unique to IgL were *MAP3K14* and 3q26.2 (a region not clearly associated with expression of a single gene), which only accounted for 7.4 and 4.9% of t(IgL) myeloma, respectively. Furthermore, other structural variants were also interrogated including deletions, duplications, and inversions, which showed that t(IgL) myeloma did not have a significantly different number of structural variants than other immunoglobulin-translocated myeloma (Supplementary Fig. 7c). Thus, these data suggest that the unique pathologic effects of IgL translocation are directly related to the IgL locus and not necessarily to a gene dysregulated by IgL transposition.

**Fig. 6:**
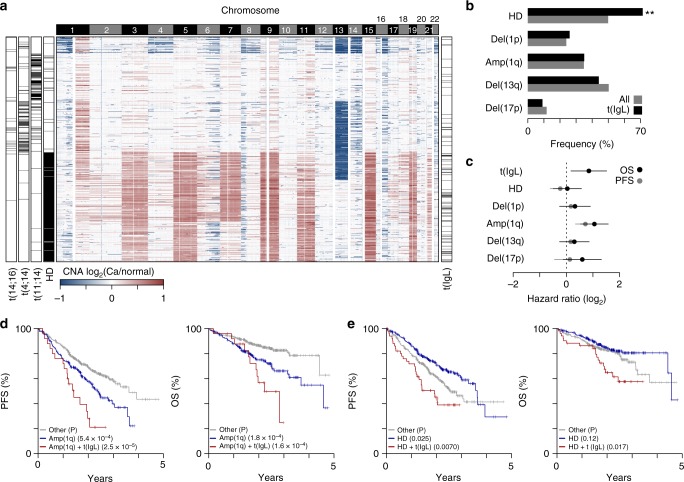
IgL translocation co-occurs with hyperdiploid disease. **a** Heatmap of copy number alterations (CNA) in 777 newly diagnosed myelomas (rows) with genomic location (columns; 100 kb bins) labelled by chromosome (top). Hyperdiploid (HD) disease, and t(11;14), t(4;14), and t(14;16) translocations are annotated (left) as well as t(IgL) (right). **b** Frequency of common CNA in all newly diagnosed myeloma (gray) and myelomas with t(IgL) (black). **P < 0.01, Fisher’s exact test. **c** Progression-free (PFS; gray) and overall survival (OS; black) hazard ratios determined by multivariate survival analysis of common CNA and t(IgL) with 95% confidence intervals shown. **d** Kaplan-Meier survival analysis of patients with amplification of 1q [amp(1q)] (blue), without amp(1q) (gray), or both amp(1q) and t(IgL) (red). **e** Same as part **d** except shown for HD. *P*-values denote the survival difference between the specified CNA (blue) and the CNA + t(IgL) (red) relative to the ‘other’ group as determined by a Cox proportional hazards Wald’s test. Source data are provided as a Source Data file

Common CNA were annotated for 777 newly diagnosed myelomas with high confidence copy number calls revealing a bifurcation in samples, where approximately half (*N* = 388) showed aneuploidy of most odd numbered chromosomes or hyperdiploidy, which was mostly mutually exclusive with IgH translocations t(11;14), t(4;14), and t(14;16) as previously reported^27^ (Fig. 6a). IgL-translocated myeloma was more commonly associated with hyperdiploid disease, but had similar frequencies of del(17p), del(1p), amp(1q), del(13q) as non-t(IgL) myelomas (Fig. 6b). Multivariate survival analysis indicated that t(IgL) was prognostic of poor PFS and OS even when accounting for all the other common CNA, some of which have themselves been independently associated with poor prognosis^28^ (Fig. 6c). For instance, patients with t(IgL) and amp(1q) myeloma had a median PFS and OS of just 1.4 and 2.2 years, respectively (Fig. 6d). Notably, patients with hyperdiploid disease generally experience better outcomes^28^, but this was not the case for the subset of hyperdiploid patients with t(IgL), who had significantly worse outcomes (Fig. 6e). These data identify IgL translocation as an independent marker of poor prognosis and further suggest that a significant fraction (15%) of patients diagnosed with hyperdiploid myeloma are being misclassified due to the co-occurrence of an IgL translocation.

### The IgL locus is a super-enhancer bound by Ikaros

The above data suggest that a factor intrinsic to the IgL locus may be mediating this disease aggressiveness, and/or that patients harboring t(IgL) are differentially responsive to therapy. Indeed, the IgL locus has previously been identified as one of the strongest super-enhancers in the myeloma cell line MM.1S as indicated by the levels of histone 3 lysine 27 acetylation (H3K27ac), or MED1 and BRD4 occupancy^29^. The IgL enhancer is also known to be bound by the transcription factors MYC and IKZF1 in other cell types^30^. This phenomenon is notable as IMiDs, which are commonly used as a front-line treatment for myeloma, target IKZF1 for CRBN-mediated ubiquitination and proteasomal degradation^18,19^. Thus, to test the hypothesis that IKZF1 regulates IgL we performed IKZF1 ChIP-seq in three myeloma cell lines including ARP-1 (IgK-expressing), MM.1S (IgL-expressing), and RPMI-8226 (IgL-expressing and translocated) (Supplementary Fig. 8a). These data indicated that IKZF1 is bound throughout the IgL locus in all three myeloma cell lines. In fact, the level of IKZF1 occupancy at the IgL locus was higher than at either the IgH or IgK locus regardless of light chain expression status (Supplementary Fig. 8b). Using an analysis analogous to that used to define super-enhancers, we found greater IKZF1 occupancy clustered across a larger region at the IgL locus relative to nearly any other locus in all three cell lines regardless of light chain expression status (Supplementary Fig. 8c). These data indicate that the IgL locus contains multiple strong enhancer elements that are bound by IKZF1.

### t(IgL) patients may not benefit from IMiD-containing regimens

**Fig. 7:**
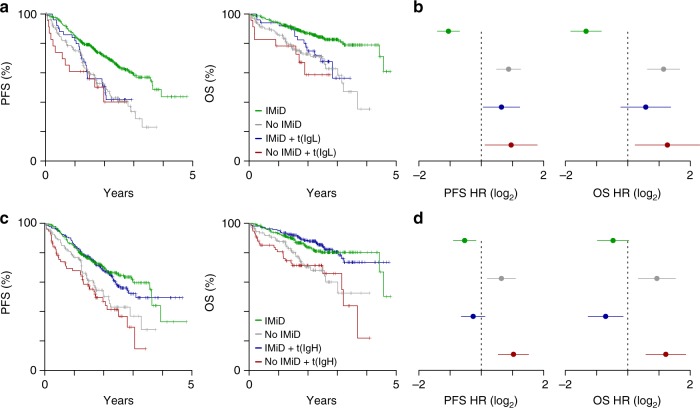
IgL translocated myelomas do not benefit from IMiDs. **a** Progression-free (PFS; left) and overall survival (OS; right) Kaplan-Meier curves for non-t(IgL) patients with (green; *N* = 463) or without (gray; *N* = 164) front-line IMiD containing therapy (PFS *P* = 3.3 × 10^−7^; OS *P* = 1.1 × 10^−6^), as well as t(IgL) patients with (blue; *N* = 51) and without (red; *N* = 23) front-line IMiD therapy (PFS *P* = 0.51; OS *P* = 0.19). **b** PFS (left) and OS (right) hazard ratios (HR) for the populations defined in part **a**. **c** PFS and OS as in part **a** except comparing non-t(IgH) patients with (green; *N* = 301) and without (gray; *N* = 111) front-line IMiD therapy (PFS *P* = 7.4 × 10^−4^; OS *P* = 1.7 × 10^−3^) as well as t(IgH) patients with (blue; *N* = 213) and without (red; *N* = 76) front-line IMiD therapy (PFS *P* = 1.5 × 10^−4^; OS *P* = 8.0 × 10^−5^). **d** PFS (left) and OS (right) hazard ratios (HR) for the populations defined in part **c**. *P*-values were calculated using a Cox proportional hazards Wald’s test. Hazard ratios in parts **b** and **d** were calculated relative to all other patients and 95% confidence intervals are shown. Source data are provided as a Source Data file

The high levels of IKZF1 occupancy at the IgL locus noted above raised the possibility that the IgL enhancer may be resistant to IMiD-based IKZF1/3 depletion, and that this might be one factor contributing to the poor outcomes of t(IgL) patients. We therefore examined the outcome of t(IgL) patients in the context of front-line IMiD-containing therapy. This analysis indicated that t(IgL) patients showed a similar poor PFS and OS regardless of whether or not they received an IMiD-containing treatment (Fig. 7a, compare blue and red lines). This is in contrast to non-t(IgL) patients who derive clear benefit from treatment with IMiD-containing regimens (Fig. 7a, compare gray and green lines). Indeed, a significant survival benefit was provided by IMiD-containing regimens for non-t(IgL) patients (PFS *P* = 3.3 × 10^−7^; OS *P* = 1.1 × 10^−6^) whereas no survival benefit was realized for patients with t(IgL) who received an IMiD-containing therapy (PFS *P* = 0.51; OS *P* = 0.19). This difference was reflected in significantly higher PFS and OS hazard ratios for t(IgL) patients regardless of treatment with a front-line IMiD-containing therapy whereas non-t(IgL) patients had a reduced hazard ratio if they received an IMiD-containing treatment (Fig. 7b). In contrast, patients with IgH translocations who received IMiD-containing therapies had a significantly lower hazard ratio and did significantly better than t(IgH) patients that did not (Fig. 7c, d; PFS *P* = 1.5 × 10^−4^; OS *P* = 8.0 × 10^−5^). Importantly, bootstrap sampling of t(IgH) patients based on the number of t(IgL) patients indicated that t(IgH) patients who received IMiD-based regimens had a better PFS than t(IgL) patients (PFS *P* = 0.045). Thus, the lack of IMiD benefit observed in t(IgL) patients is not simply due to the smaller number of t(IgL) patients. Together, these data suggest that IMiDs are less effective against t(IgL) myeloma than other myelomas.

## Discussion

Here, we present the first comprehensive catalogue of translocations in newly diagnosed multiple myeloma using whole genome sequencing on 795 tumor specimens with matched germline controls. This analysis identified 20 translocation hotspots and 11 recurrent translocations. As expected, the most common translocated region was IgH, which was primarily juxtaposed to known myeloma oncogenes *CCND1*, *WHSC1*, *MYC*, and *MAF*. IgH translocation were mostly clonal and breakpoints commonly occurred in the switch regions of the constant chains, implicating an error in class switch recombination in germinal center B cells, consistent with previous observations^11^. However, this was not the case for IgH-MYC translocations, which primarily marked sub-clonal cell populations and were located in extragenic regions further 3′ of the IgH locus. This suggests that IgH-MYC translocations occur by a different mechanism and is consistent with the suggestion that MYC translocations are secondary events in myelomagenesis^21^, albeit common secondary events. Additionally, t(11;14) breakpoints showed only marginal enrichment for class switch recombination regions, as several translocations also occurred in the VD and extragenic regions, similar to previous reports^31^. This suggests that t(11;14) myelomas occur through at least two different mechanisms that may have distinct disease biologies.

MYC translocations and CNA were present in 23% and 32% of newly-diagnosed patients, respectively, with a significant overlap between the two. Indeed, 85% of cases that harbored MYC translocations also contained a MYC CNA, and these were primarily amplifications that abutted the translocation breakpoint. These amplifications are reported to result from large inversions at reciprocal translocation breakpoints^20^. Both amplifications and translocations resulted in commensurate upregulation of MYC as compared to other myelomas, but even those without a MYC structural variant, expressed MYC at a substantial level, supporting the notion that MYC overexpression is a common feature of myeloma^13^. The one exception is those myelomas with loss-of-function mutations in the MYC binding partner MAX, which express MYC at substantially lower levels^32^. As a whole, newly diagnosed patients that presented with MYC translocations had an outcome similar to those that did not have a MYC translocation, consistent with previous studies^33^. However, patients with IgL-MYC translocations experienced a poor prognosis, despite having similar baseline *MYC* expression as other MYC translocations. Given the complex nature of the structural variations at the MYC locus, one potential explanation is that translocation of the IgL enhancer may influence other genes or RNA species in and around the MYC locus, and this contributes to differential outcomes. However, this regulation would need to be unique to IgL translocations as IgH and IgK translocations at the MYC locus do not result in decreased survival. Therefore, an alternative explanation for these seemingly non-intuitive findings is that the baseline gene expression of *MYC* may fail to predict the therapeutic effect on oncogene regulation, i.e., different enhancers may drive the same level of MYC expression but may be differentially susceptible to therapeutic disruption of MYC expression. This inference is supported by the observation that t(IgL) myeloma patients had an adverse clinical course despite indistinguishable patient characteristics, clinical features, treatment regimens, and mutations as the entire cohort. These data support the notion that the IgL locus, when translocated to an oncogene, may have distinct effects from that of the IgH or IgK enhancers.

Several studies have reported IgL translocations^13,34–37^, but to our knowledge this is the first study to report a frequency approaching 10% in newly diagnosed myeloma. The high frequency of IgL translocations was somewhat surprising given that 65% of myelomas express IgK, which was only translocated in 4.5% of patients. Interestingly, the RNA-seq data here indicate that t(IgL) myeloma has the expected ratio of light chain expression with two-thirds expressing IgK and one-third expressing IgL, whereas 100% of t(IgK) myeloma express IgK (Supplementary Fig. 9a–c). These observations are explained by IgK VJ recombination, which in most cases somatically deletes the IgK 3′ enhancer when a productive allele is not recombined^38^. Thus, roughly half of IgK expressing B cells, and most of IgL-expressing B cells have somatically deleted at least one copy of the IgK enhancer. Indeed, copy number data clearly indicates that the majority of IgL-expressing myelomas have somatically deleted the IgK 3′ enhancer (Supplementary Fig. 9d, top). Conversely, the IgL enhancer, which is one of the largest enhancers in B cells, plasma cells, and myeloma, appears to be active regardless of whether or not a productive IgL is expressed, as indicated by chromatin accessibility and IKZF1-occupancy in IgK- and IgL-expressing myeloma cell lines (Supplementary Fig. 9d, bottom). This constitutive activity partially explains why IgL translocations are more common than IgK.

Although IgL translocations including IgL-MYC, did not coincide with other small insertions/deletions, mutations, or patient clinical characteristics, t(IgL) was more common in myelomas exhibiting hyperdiploidy. The poor prognosis of patients with IgL-MYC translocations is independent of hyperdiploidy and other major CNAs and structural variants. While 41% (*N* = 32) of patients with IgL-translocations were IgL-MYC, the remaining were transposed throughout the genome to a multitude of regions with the most common loci including *MAP3K14* (*N* = 6), 3q26.2 (*N* = 4; proximal to *SEC62* and *GPR160*), and *CCND2* (*N* = 3). Subsequently, we conclude that IgL-MYC translocations are likely prognostic of poor outcome, whereas the rarity of other types of IgL translocations makes it currently impossible to assess which of these events also contribute to poor outcome. Identification of IgL-MYC translocated myeloma will require a rapid and reliable diagnostic that can be used for appropriate risk-stratification. This is particularly important as t(IgL) is rarely identified clinically, whereas FISH is routinely performed to identify hyperdiploidy, which corresponds with better prognosis^28^. As a result, IgL-MYC translocations represent an unrecognized high-risk marker prevalent in a subset of patients that are currently considered to be standard risk.

One indication of how IgL-MYC translocations may contribute to poor outcome is provided by the interaction of t(IgL) and treatment, which suggested that t(IgL) patients did not benefit from IMiD-containing regimens. The therapeutic efficacy of IMiDs may be mediated, in part, by their ability to inhibit activity of IKZF1/3 regulated enhancers, including those juxtaposed to oncogenes by translocation. While translocation of the immunoglobulin loci is a common mode of oncogene overexpression in myeloma, the IgL locus is unique in that, regardless of IgL expression, the IgL enhancers appear to be very active and bound by some of the highest levels of IKZF1 in the myeloma epigenome. This might render the IgL locus particularly insensitive to IMiD-based depletion of IKZF1 and may explain why t(IgL) patients do not benefit from IMiD treatment. It is important to note that we cannot rule out other potential causes for the poor prognosis of patients with t(IgL) myeloma such as refractory responses to other therapies or differences in the micro-environment or immune response. Furthermore, these findings will need to be validated in a clinical trial setting where treatment options can be standardized. It will also be important to more completely understand how IKZF1 regulates the IgL enhancer in myeloma and in the context of translocation and IMiD-mediated degradation. Recent work has shown the IgL locus to be one of the largest super-enhancers as measured by MED1 or BRD4 occupancy in MM.1S cells^39^, suggesting several distinct transcriptional regulators may be mediating IgL-driven oncogene expression in t(IgL) myeloma. Thus, it will also be important to understand the efficacy of emerging transcriptional therapeutics such as BET inhibitors^40^ or degraders^41^ that abolish BRD4 as well as other small molecule inhibitors that target the transcriptional machinery driving oncogene expression. The data herein provide motivation for determining the efficacy of such transcriptional regulators in the context of t(IgL) myeloma, but also a rationale for the better understanding of cis-regulatory factors affecting transcription at translocated loci. This will not only require an understanding of the combinatorial effects of the trans-acting molecular machinery, but also a cartography of the chromatin structure and epigenetic mechanisms known to influence plasma cell fate^42,43^, as well as enhancer function in the context of translocation breakpoints. Such results will ultimately need to be placed in the context of multicellular organisms and bone marrow micro-environmental cues to help identify drug targets with high specificity for the transcriptional program and genetic architecture of myeloma.

## Methods

### CoMMpass

Work complied with all relevant ethical regulations for work with human participants and IRB approval for patients participating in the CoMMpass study was obtained by Emory IRB. Use of CoMMpass data (interim analysis 11) was approved by the data access use committee and downloaded from dbGaP (phs000748.v6.p4).

### Whole genome sequencing

Long-insert whole genome library preparation and sequencing was performed by the Translational Genomics Institute (TGen) using 200-1,100 ng of DNA. DNA was sonicated (Covaris) to an average size of 900 bp. Libraries were prepared with either the TruSeq DNA Sample Prep (Illumina) or Hyper Prep (Kapa Biosystems) following the manufacturer’s instructions using AMPure beads (Invitrogen) for clean-up. Adapter ligated libraries were run on a 1.5% agarose gel and extracted by hand or with an Extracta-gel-extractor (USA Scientific). Size-selected libraries were purified with DNA Gel Extraction spin columns (Bio-Rad) prior to library amplification. Library size was assessed on a Bioanalyzer (Agilent) and quantified using the Qubit dsDNA HS assay (Invitrogen) and on a TapeStation (Agilent). Paired-end sequencing was performed using a HiSeq2000 or HiSeq2500 (Illumina) with either v3 or v4 chemistry and 86 bp reads. Raw sequencing data was extracted from BCL files using BCL2FASTQ v2.17.1 to extract FASTQ files.

### Whole genome sequencing analysis

Sequencing FASTQ files were mapped to a modified version of the human genome GRCh37 using BWA (v0.7.8)^44^. The specific reference genome was phase 2 of the 1000 Genomes project^45^ plus contigs from ribosomal DNA and cancer-related viruses (U13369.1, FR872717.1, AF092932.1, NC_001526.2, NC_001357.1, NC_003977.1, NC_004102.1, NC_009823.1, NC_009824.1, NC_009825.1, NC_009826.1, NC_009334.1, NC_006273.2, NC_011800.1, NC_001806.1) as well as sequence from the External RNA Controls Consortium (ERCC)^46^. Aligned BAM files were sorted with SAMtools (v0.1.19)^47^, recalibrated with GATK (v3.1.1)^48^, and putative PCR duplicates were marked with Picard (v1.111). Tumor-specific indel realignment was performed relative to normal samples collected at the same time from the same patient using GATK (v3.1.1).

### Determination of structural variants

Structural variants were determined using DELLY (v0.7.6)^49^ with the following filter module options: altFrac = 0.1, ratioGeno = 0.75, coverage = 5, controlContamination = 0, minSize = 500, maxSize = 500000000. DELLY BCF files were converted to VCF files using BCFTOOLS and VCF files were parsed, formatted, and annotated using custom R scripts available upon request. DELLY translocations were subject to further quality control that included homology searches of 1 kb on either side of the translocation break point between the two transposed regions where translocations with homology of 80% or more in any 100 bp window were removed as false positives. Translocations were compared to the ENCODE^30^ 20 bp mappability tracks and those translocations that had an average mappability of less than 20% across 1 kb on either side of the translocation breakpoint were also removed. Finally, translocations were visually inspected and compared to the mapped reads in BAM files resulting in elimination of translocations in certain regions with sequencing anomalies (Supplementary Data 6). The VAF of each translocation was determined as the ratio of reads spanning the translocation relative to the total number of reads spanning the translocation breakpoint.

### Identification of translocation hotspots

Commonly translocated regions were identified using a 1 Mb window incremented 500 kb across each chromosome. Contiguous 1 Mb regions translocated at a frequency of 1% or more were stitched together. Common translocations were identified by calculating the frequency of any given translocation type within 1 Mb of the two chromosomal breakpoints.

### Translocation visualization

R / Bioconductor (v3.4.3)^50^ was used to generate circos plots with the RCircos package (v1.2.0)^51^. Heatmaps of translocation occurrence by sample were generated with the ‘image’ function in a method similar to the ‘heatmap’ function of the stats package. Plots of translocations across genomic regions were made as previously described^42^ and translocations were represented by a region covering 1 kb of each side of the breakpoint or estimated breakpoint.

### RNA-seq

RNA-seq libraries were constructed with the TruSeq RNA Library Prep Kit v2 (Illumina) by TGen, which yields unstranded mRNA libraries. 150–2000 ng of RNA, which had an RNA integrity number (Agilent Bioanalyzer) of 8 or higher was used for starting material. RNA libraries were amplified for 8–10 cycles and then sequenced on an Illuina HiSeq2000 or HiSeq2500 using v3 or v4 chemistry and 82 bp paired-end reads.

### RNA-seq analysis

RNA-seq reads were aligned to the same GRCh37 genome as whole genome sequencing data using STAR (v2.3.1)^52^ with the GRCh37.74 gtf file to guide splice junction identification. Coverage was determined with HTSeq (v0.6.0)^53^ and FPKM normalization was done using R / Bioconductor without the use of IMGT-defined immunoglobulin genes, which were analyzed separately. IgH constant chain expression was determined by the most highly expressed constant region so long as that was ≥5000 FPKM. Likewise, light chain expression was determined as the highest expression (measured by FPKM) of the cumulative variable, joining, and constant regions for IgK and IgL. Expression of individual genes were plotted with R ‘boxplot’ function with outliers plotted using the beeswarm package (v0.2.3). Ad-hoc differences in gene expression for translocated genes was determined with the Mann-Whitney U-test for pairwise comparisons or analysis of variance with Tukey’s post hoc correction for multiple comparisons.

Gene expression subtypes were determined using the ‘ConcensusClusteringPlus’ (v1.42.0)^54^ in R/Bioconductor using log_2_(FPKM + 1) transformed data that was gene normalized for 1000 iterations using a hierarchical clustering algorithm and a Pearson distance metric performed on 2 to 20 consensus groups. Increasing the consensus groups beyond 7 explained only minimal variation, thus 7 was chosen. Genes uniquely expressed in each consensus group were determined by comparing each group to all other samples using edgeR^55^ with an FDR ≤ 0.01 as were genes differentially expressed between t(IgL) myeloma and other myelomas. t(IgL) differentially expressed genes were further confirmed by restricting the analysis to only samples with hyperdiploidy and by performing analysis with a co-variate for gene expression subtype determined by consensus clustering. Gene set enrichment analysis (GSEA)^25^ was performed using a pre-ranked list determined by the −log_10_(*P*-value) × sign(fold-change) against all curated gene lists in MSigDB (v6.1) and for those from Zhan et al.^24^ to match gene expression subtypes to those previously identified.

### Exome-sequencing

Exome sequencing was performed by TGen on DNA from CD138+ myeloma cells and peripheral blood references samples for all individuals. DNA was fragmented with a Covaris and library preparation was performed with either TruSeq exome kit (Illumina) or with the HyperPrep kit (Kapa) in combination with the Sureselect V5 + UTR exome capture kit (Agilent). Data were mapped to the same reference genome (GRCh37, plus contigs defined above) using BWA (v0.7.8)^44^. As with the whole genome long-insert sequencing, aligned BAM files were sorted with SAMtools (v0.1.19)^47^, recalibrated with GATK (v3.1.1)^48^, and putative PCR duplicates were marked with Picard (v1.111).

### Mutational analysis

Somatic SNV and INDELS were based on calls from Seurat (v2.6)^56^, Strelka (v1.013)^57^, and MuTect (v1.1.4)^58^. Final mutations were determined as those that were called by at least two of the three aforementioned tools. Mutations were annotated to GRCh37.74 protein coding genes and immunoglobulin genes were excluded from analysis due to somatic hypermutation. Synonymous and non-synonymous mutations were determined using the ‘locateVariants’ and ‘predictCoding’ functions of the ‘VariantAnnotation’ (v1.24.5) package^59^. Mutational differences between t(IgL) and non-t(IgL) myelomas were assessed using Fisher’s exact test with an FDR correction for all mutations present at frequency of ≥4% of the population.

### CNA analysis

CNA were determined separately for exome sequencing and whole genome long-insert sequencing using the TGen tool tCoNut (https://github.com/tgen/tCoNuT). Exome-sequencing derived CNA were used to define large cytogenetic abnormalities including hyperdiploidy, del(1p), amp(1q), del(13q), and del(17p). CNA gains and losses were defined as log_2_ CNA ratio of myeloma to normal of ≥0.2 and ≤0.2, respectively. The regions in Table 1 were used to define common myeloma CNA based on the average CNA segmentation call for the region.

**Table 1:** Regions used to define common copy number alterations

Name	Region
del(1p)	1p22
amp(1q)	1q21
del(13)	13q14
del(17)	17p13
Hyperdiploidy^a^	3p22, 3q21, 5p15, 5q31, 7p14, 7q22, 9p13, 9q33, 11p15, 11q23, 15q15, 15q26, 19p13, 19q13, 21q21, 21q22

^a^Hyperdiploidy required concordant gains of both areas on a given chromosome for 4 chromosomes

Common copy number alterations are listed on the left and the region used to define alterations is listed on the right

CNA analysis of global chromosome changes (i.e., Fig. 6a) was done by calculating the average CNA log_2_ ratio for 100 kb bins across the genome and clustering was performed using ConsensusClusterPlus^54^. Intergenic CNA, including those at the MYC, IgK, and IgL loci, were derived from whole genome long-insert sequencing data and binned into 100 bp increments.

### Survival analysis

Survival analysis was conducted using in R using the ‘survival’ (v2.41-3) package. Differences in PFS and OS were determined using a cox proportional hazards regression fit to either a continuous (e.g., the number of deletions, duplications, inversions, or translocations) or discrete (e.g., t(IgL) versus other) variable. *P*-values were calculated using a Wald’s test. When more than two discrete variables existed a *P*-value of differences between all groups was first calculated followed by pairwise comparisons and FDR correction. Hazard ratios associated with translocations, mutations, and other clinical variates were also calculated using a cox proportional hazards regression and 95% confidence intervals are shown. Bivariate analysis was performed for the most common mutations in combination with t(IgL) and multivariate analysis was conducted with clinically relevant parameters and t(IgL) as well as common CNA and t(IgL). Bootstrapping of differences in outcome used 1000 permutations and compared the permuted PFS and OS hazard ratios as compared to the actual hazard ratio. The comparison of IMiD survival benefit in t(IgL) versus t(IgH) sampled t(IgH) patients according to the number of t(IgL) patients.

### Cell culture

Myeloma cells lines were cultured at a density of 0.2–1.0 × 10^6^ cells per mL in RPMI 1640 (Corning: 14-030-CV) with 10% FBS, 1% Pen-strep (Corning: 30-002-CI), 1% L-glutamine (Corning: 25005CI), and 1% HEPES (Corning: 25-060-CI). Cells were purchased from the American Type Culture Collection, the Japanese Collection of Research Bioresources, or Deutsche Sammlung von Mikroogransmen und Zellkulturen, except for ARP-1, which was obtained from the source^60^. Cell lines were validated using sequencing and phenotypic characterization.

### IKZF1 ChIP-seq

IKZF1 ChIP-seq was performed using 20 × 10^6^ cells, which were cross-linked for 20 min at room temperature with a final concentration of 1% formaldehyde solution (5 mM Hepes – KOH pH 7.5; 10 mM NaCl; 100 μM EDTA pH 8; 5 μM EGTA pH 8; 1% formaldehyde) followed by a 5 min quench with 125 mM glycine. Cross-linked cells were washed twice in PBS. 100 μl of Dynal protein G magnetic beads (Sigma) were washed 3× for 5 min at 4 °C in 0.5% BSA in PBS prior to blocking for ≥5 h with 0.5% BSA in PBS. Magnetic beads were bound with 10 μg of IKZF1 antibody (GeneTex, GTX129438).

Cross-linked cells were lysed with lysis buffer 1 (50 mM HEPES pH 7.3; 140 mM NaCl; 1 mM EDTA; 10% glycerol; 0.5% NP-40; 0.25% Triton X-100; protease inhibitors) and resuspended in sonication buffer (20 mM Tris-HCl pH 8.0, 150 mM NaCl, 2 mM EDTA pH 8.0, 0.1% SDS, 1% Triton X-100) prior to sonication on a E210 (Covaris). Sonicated lysates were incubated overnight at 4 °C with Dynal magnetic beads bound with antibody. Beads were washed two times with sonication buffer, one time with sonication buffer with 500 mM NaCl, one time with LiCl wash buffer (10 mM Tris pH 8; 1 mM EDTA; 250 mM LiCl; 1% NP-40) and one time with TE + 50 mM NaCl. DNA was eluted in elution buffer (50 mM Tris-HCl pH 8; 10 mM EDTA; 1% SDS) at 65 °C. Cross-links were reversed overnight at 65 °C and RNA and protein were digested using first RNase A and then Proteinase K at 37 °C. DNA was purified with phenol chloroform extraction and ethanol precipitated prior to library preparation using Rubicon ThruPLEX DNA-seq (Rubicon Genomics). Libraries were sequenced to a depth of at least 50 × 10^6^ reads on an Ion Proton (Thermo Fisher) sequencer.

### ATAC-seq

ATAC-seq was performed on 50,000 viable sorted myeloma cells similar to previously described^43,61,62^. Briefly, cells were pelleted at 500 × *g* for 10 min at 4 °C and resuspend in ice-cold nuclei-lysis buffer (10 mM Tris pH 7.4, 10 mM NaCl, 3 mM MgCl_2_, 0.1% IGEPAL) and centrifuged at 500 × *g* for 30 min at 4 °C. Nuclei were resuspended in 22.5 μl of tagmentation DNA buffer (Illumina) with 2 μl tagmentation enzyme (Illumina) at 37 °C for 60 min. Proteins were digested with 2 μg of proteinase K at 40 °C for 60 min. Tagmented DNA was isolated with two rounds of negative (0.6×) and positive (1.2×) size selection with SPRI beads (PureBeads, Kapa Biosystems). ATAC-seq libraries were amplified 12 times with Hifi Polymerase (Kapa Biosystems) and quantitated by qPCR (Kapa Biosystems) and high sensitivity bioanalyzer (Agilent). Sequencing was performed on a NovaSeq 6000 (Illumina) using 150 bp paired-end at the New York Genome Center.

### ATAC-seq and ChIP-seq analysis

ChIP-seq FASTQ files were mapped to the same GRCh37 genome used above for CoMMpass data using bowtie 2 (v2.2.6)^63^. Mapped SAM files were converted to BAM files and putative PCR duplicates were marked using SAMtools (v1.7)^47^. IKZF1 enriched regions were determined using MACS2 (v2.1.0.20151222)^64^ using default parameters and a *q*-value of 0.01. Fragment size was estimated using the R package ‘chipseq’ (v1.28.0) and reads were extended to the estimated fragment size for visualization using the R package ‘rtracklayer’ (v1.38.3)^65^. Super-enhancer analysis was performed using custom R code in a manner analogous to that done previously^39^. Briefly, this involved stitching together enriched regions that were within 15 kb of each other and did not overlap a promoter region defined as 2.5 kb upstream of the TSS for GRCh37.74 defined promoters. Regions were then ranked by occupancy measured as reads per million (RPM) and regions that were past the inflection point were considered super-enhancers.

### Reporting summary

Further information on experimental design is available in the Nature Research Reporting Summary linked to this article.

## Supplementary information

Supplementary Information

Description of Additional Supplementary Files

Supplementary Data 1

Supplementary Data 2

Supplementary Data 3

Supplementary Data 4

Supplementary Data 5

Supplementary Data 6

Reporting Summary

Source Data

## Data Availability

CoMMpass data is deposited in dbGaP (phs000748.v6.p4) and summarized data can be accessed at https://research.themmrf.org/. ATAC-seq data is available under GEO accession GSE121912. ChIP-seq data are available under GEO accession GSE128024. The source data underlying Figs. 1, 2, 3a, b, g, h, 4a, b, e, 5, 6, and 7 are provided as a Source Data file.

## Code availability

All code is available upon request.
