# Supplementary material for: Multiple myeloma immunoglobulin lambda translocations portend poor prognosis
Source: Nat Commun. 2019 Apr 23;10:1911. doi: 10.1038/s41467-019-09555-6 (PMC6478743; doi:10.1038/s41467-019-09555-6)
Supplement: Supplementary file 1 — Supplementary Information [file 41467_2019_9555_MOESM1_ESM.pdf]

Supplementary Material for:

Multiple myeloma immunoglobulin lambda translocations portend poor prognosis

Benjamin G. Barwick[1,2,3], Paola Neri[4], Nizar J. Bahlis[4], Ajay K. Nooka[1,3], Madhav V. Dhodapkar[1,3], David L. Jaye[3,5], Craig C. Hofmeister[1,3], Jonathan L. Kaufman[1,3], Vikas A. Gupta[1,3], Daniel Auclair[6], Jonathan J. Keats[7], Sagar Lonial[1,3], Paula M. Vertino[2,3*#], Lawrence H. Boise[1,3*]

[1]Department of Hematology and Medical Oncology, Emory University School of Medicine, 1365 Clifton Rd. NE, Atlanta, GA 30322

[2]Department of Radiation Oncology, Emory University School of Medicine, 1701 Uppergate Drive, Atlanta, GA 30322

[3]Winship Cancer Institute, Emory University, 1365 Clifton Rd, Atlanta, GA 30322

[4]Charbonneau Cancer Research Institute, University of Calgary, 3330 Hospital Drive, Calgary, AB, T2N 4N1

[5]Department of Pathology and Laboratory Medicine, Emory University School of Medicine, Atlanta, GA 30322

[6]Multiple Myeloma Research Foundation, 383 Main Avenue, 5th Floor, Norwalk, CT 06851

[7]Translational Genomics Research Institute, 445 North Fifth Street, Phoenix, AZ, 85004

*Address correspondence to:
Paula M. Vertino, Ph.D.
pvertin@emory.edu
(404) 778-3119
and
Lawrence H. Boise, Ph.D.
lboise@emory.edu
(404) 778-4724

# Present address: Department of Biomedical Genetics, University of Rochester School of Medicine and Dentistry, Rochester, NY, 14642

Figure S1

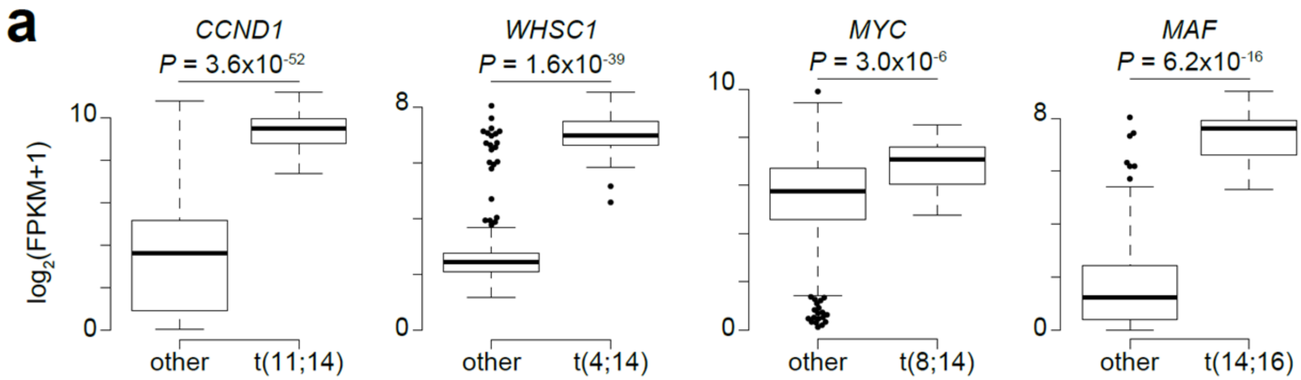

b

IgH	N	VD	CSR	Extragenic
t(11;14)	212	25 (11.8%)	160 (75.5%)	27 (12.7%)
t(4;14)	122	2 (1.6%)	119 (97.5%)	1 (0.8%)
t(14;16)	40	2 (5.0%)	34 (85.0%)	4 (10.0%)
t(8;14)	37	3 (8.1%)	8 (21.6%)	26 (70.3%)
other	148	26 (17.6%)	62 (41.9%)	60 (40.5%)
total	559	58 (10.4%)	383 (68.5%)	118 (21.1%)

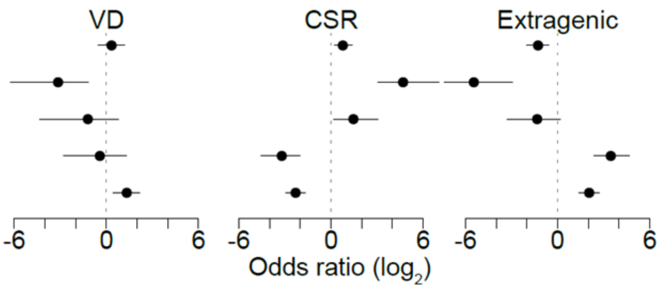

Figure S1. IgH translocations correspond with aberrant gene expression. **a** Expression of translocated genes in t(IgH) myeloma. Boxplots show the median and quartiles with the whiskers extending to the most extreme data point within 1.5 times the interquartile range. *P*-values are calculated using the Mann-Whitney U-test. **b** Table of IgH location that distinct IgH translocations occur segregated by the variable and diversity (VD), class switch recombination (CSR) (+/-2.5kb), and extragenic regions. Note: individual patients may have more than one reported translocation. The odds-ratio of each translocation type are plotted (right) with 95% confidence intervals shown.

Figure S2

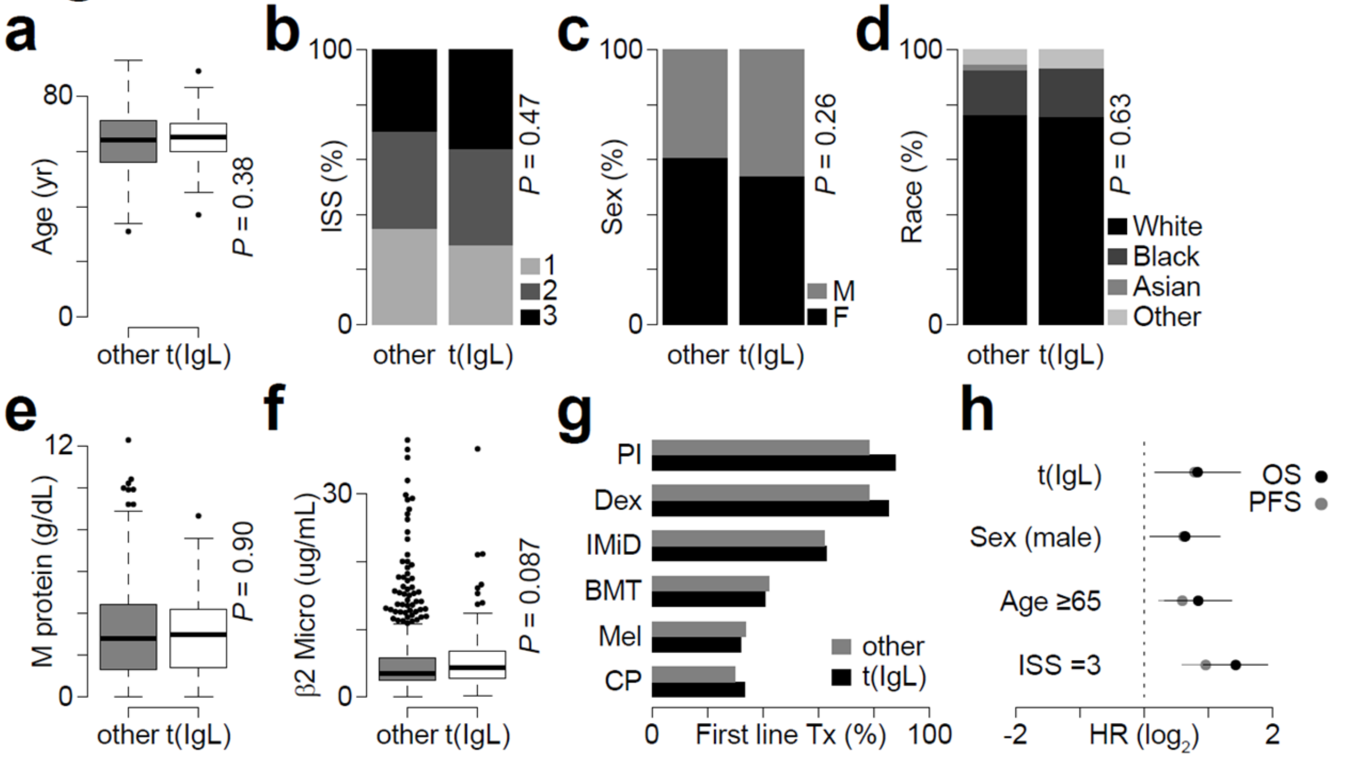

Figure S2. Patients with t(IgL) have similar characteristics and clinical features as other myeloma patients. Patient (**a**) age in years, (**b**) stage (ISS), (**c**) sex, (**d**) race, (**e**) serum M-protein, and (**f**) serum β2-microglobulin are shown for non-t(IgL) and t(IgL) patients. **g** Therapeutic agents used in treatment regimens including proteasome inhibitors (PI), dexamethasone (Dex), immunomodulatory imide drugs (IMiD), autologous bone marrow transplant (BMT), melphalan (Mel), and cyclophosphamide (CP) are shown for t(IgL) and other myelomas. **h** OS (black) and PFS (gray) hazard ratios (HR) determined by multivariate Cox proportional hazards analysis of t(IgL) with prognostic clinical factors. 95% confidence intervals are shown. *P*-values represent significance determined by Mann-Whitney U-test (**a**, **e**, **f**) or Fisher's exact test (**b**, **c**, **d**). Boxplots (**a**, **e**, **f**) show the median and quartiles with the whiskers extending to the most extreme data point within 1.5 times the interquartile range.

Figure S3

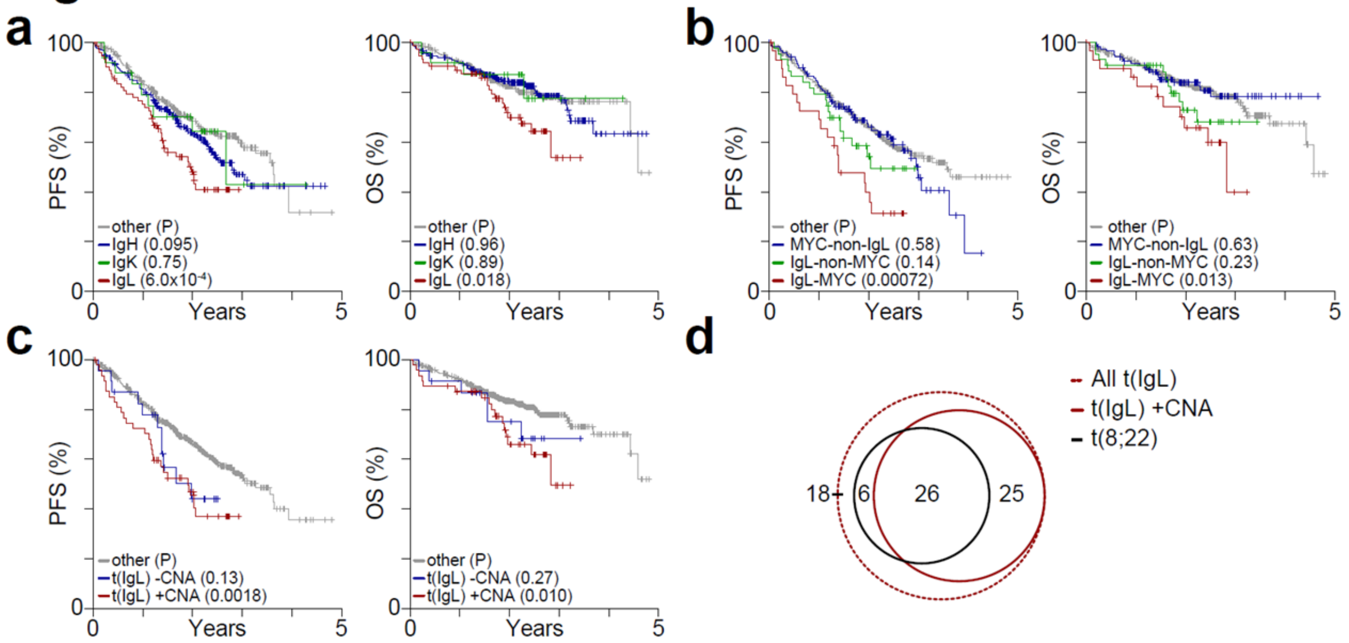

Figure S3. IgL translocations are associated with poor prognosis. **a** Progression-free (PFS; left) and overall survival (OS; right) for patients stratified by immunoglobulin translocation: other (N=383), IgH (N=304), IgK (N=30), IgL (N=78). **b** PFS (left) and OS (right) for patients stratified by IgL and MYC translocations: other (N=580), MYC-non-IgL (N=137), IgL-non-MYC (N=46), IgL-MYC (N=32). **c** PFS (left) and OS (right) for patients stratified by IgL-translocation with (N=51) and without (N=24) an IgL copy number alteration (CNA) as well as those with no IgL-translocation (N=702). **d** Venn diagram of all t(IgL) samples (dashed red), t(IgL) samples with a focal amplification (red; t(IgL) +CNA), and t(8;22) samples. All samples with structural variant data (N=795) were used in parts **a** and **b**, and all samples with structural variant and copy number data (N=777) were used in parts **c** and **d**. P-values, shown in parenthesis in parts **a-c**, were calculated using a Cox proportional hazards Wald's test and denote significant differences in survival relative to the 'other' group.

Figure S4

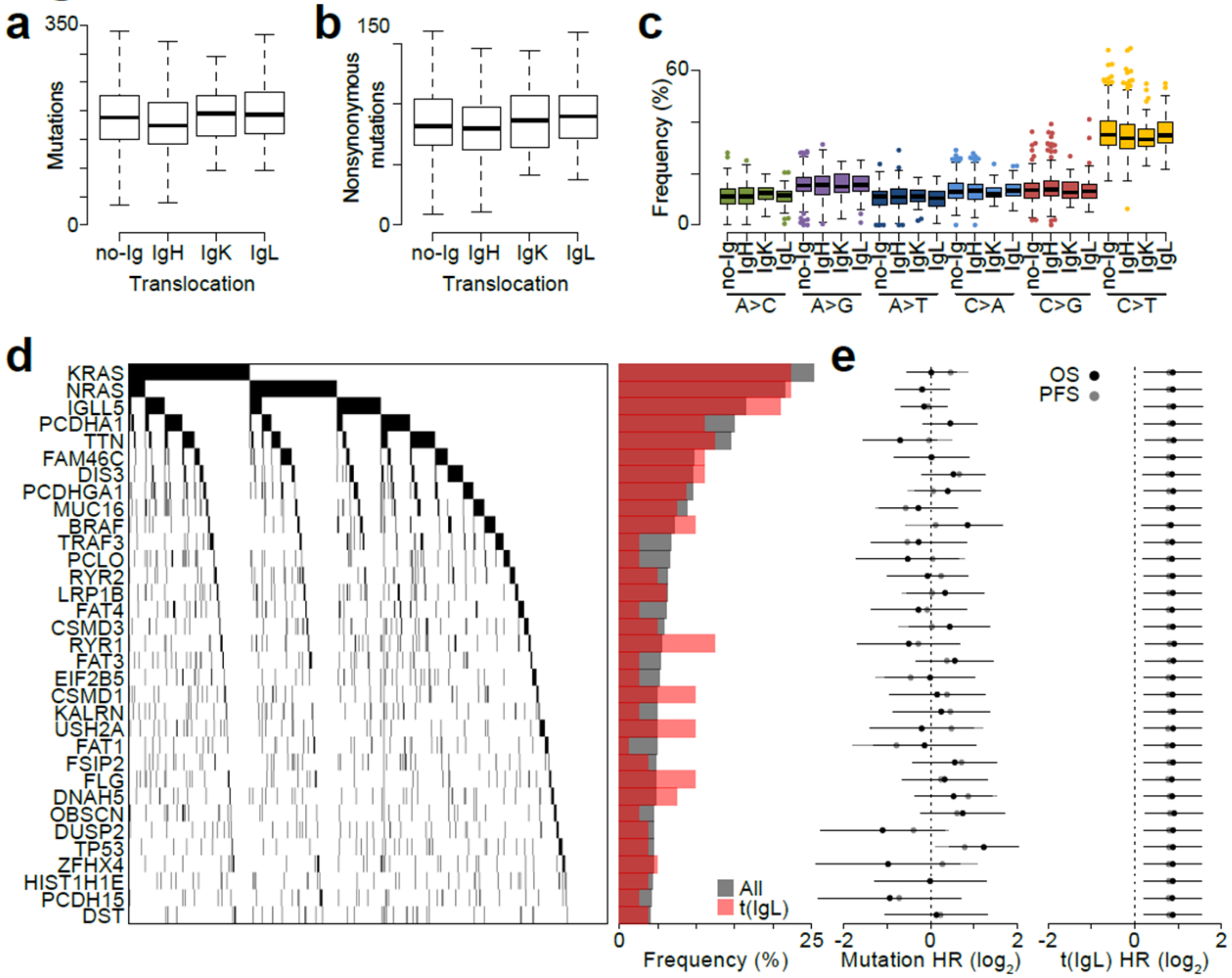

Figure S4. IgL translocations do not correspond with tumor-specific mutations. **a** Number of total mutations in newly diagnosed myelomas with no immunoglobulin translocation (no-Ig), or an IgH, IgK, or IgL translocation. **b** Number of nonsynonymous mutations in the same myelomas as part (**a**). **c** Frequency of mutational repertoire in the same myelomas as part (**a**). **d** Waterfall plot of myeloma nonsynonymous mutations for genes with a frequency at least 4% in newly diagnosed myelomas (left). The frequency of mutations for each gene is shown (right) for both the total population (gray) and t(IgL) myeloma (red). **e** Progression-free (PFS; gray) and overall survival (OS; black) hazard ratios (HR) are shown for each mutation (middle) in a bivariate analysis including t(IgL) (right). HR 95% confidence intervals of are shown. Data include all newly diagnosed myeloma samples with long-insert and exome sequencing in both tumor and normal samples (N=783), which includes patients with no-Ig translocation (N=377), IgH (N=298), IgK (N=30), and IgL (N=78) translocations. Only mutation in non-immunoglobulin regions are analyzed. Boxplots (**a**-**c**) show the median and quartiles with the whiskers extending to the most extreme data point within 1.5 times the interquartile range.

Figure S5

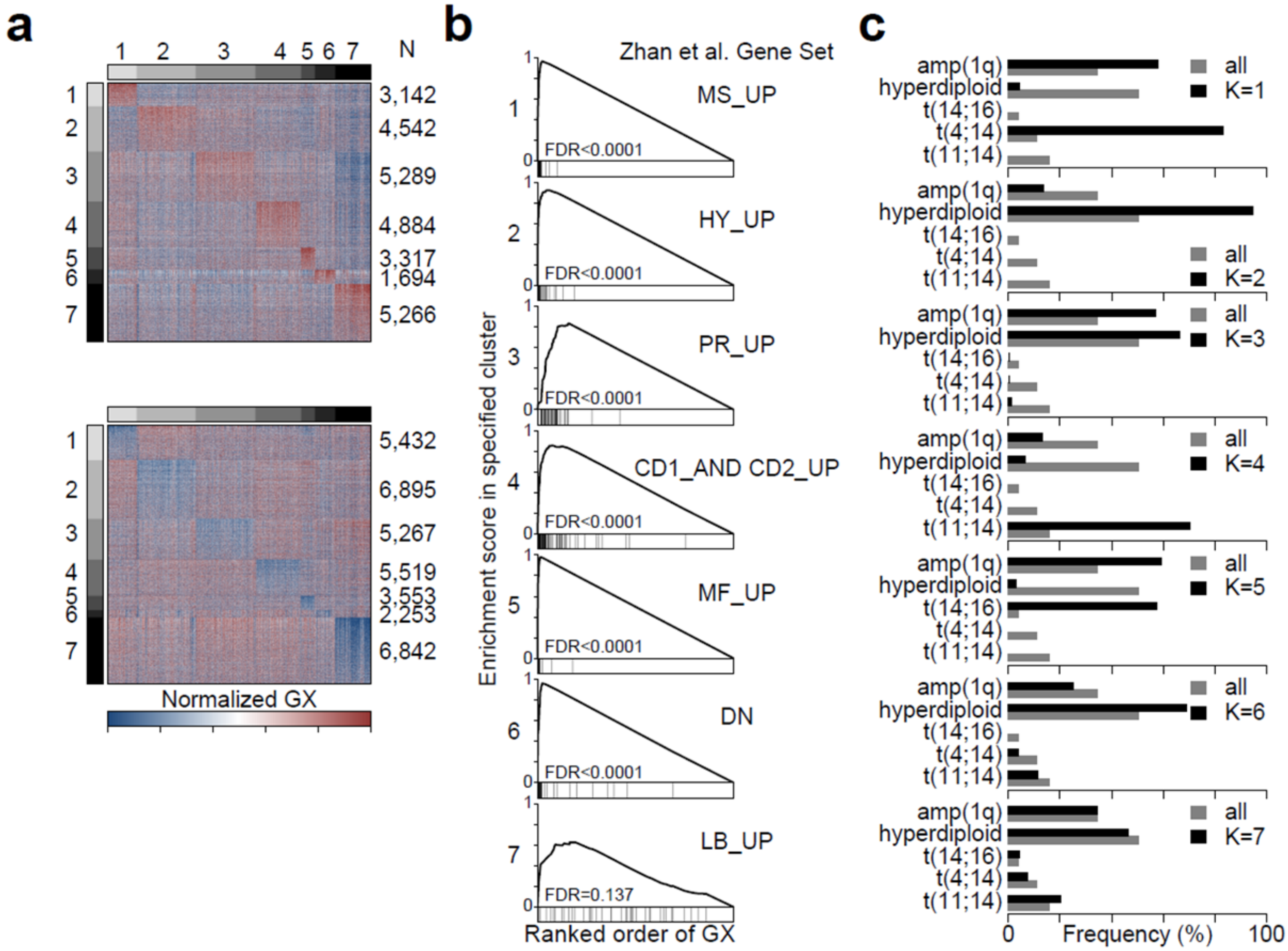

Figure S5. Myeloma gene expression subtypes. **a** Heatmap of genes up- (top) and down-regulated (bottom) in each expression subgroup. The number of genes in each category are denoted on the right. Genes found to be significantly regulated in multiple subtypes were plotted in the subtype where their significance was greatest. **b** Gene set enrichment analysis of the most enriched gene set from Zhan et al.[24]. Enrichment score is shown on the y-axis and the x-axis is a ranked order of gene expression changes from most upregulated (left) to most downregulated (right) in the given expression subtype. **c** Frequency of genetic translocations and copy number alterations in each subtype relative to total patients.

Figure S6

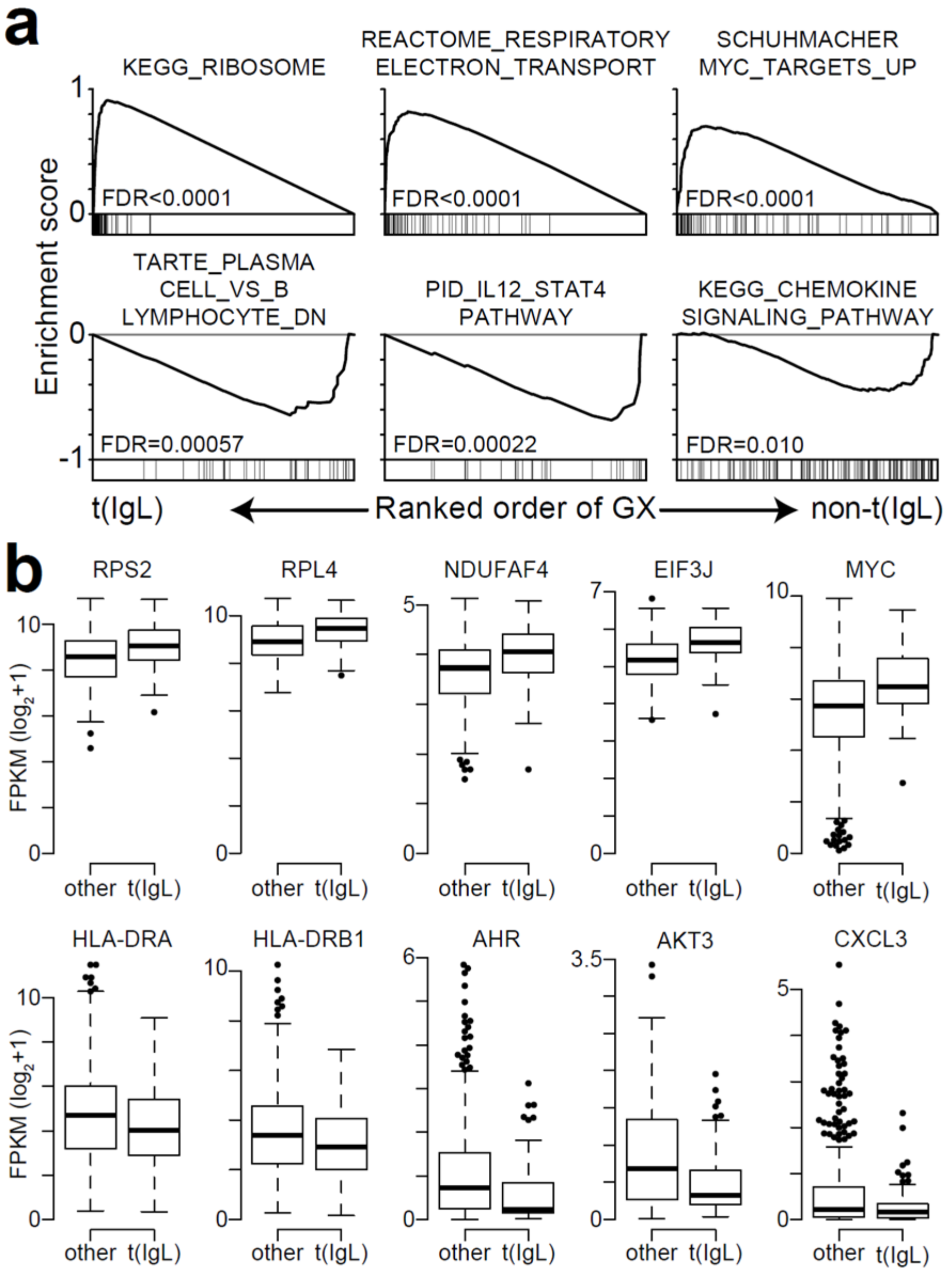

Figure S6. Gene expression changes coincident with t(IgL). **a** Gene set enrichment analysis of gene sets enriched (top) or depleted (bottom) in t(IgL) myeloma. Enrichment score is shown by the ranked order of gene expression changes from most upregulated in t(IgL) (left) to most down-regulated (right). **b** Examples of genes differentially expressed from the above genes sets including both upregulated (top) and downregulated (bottom) genes in t(IgL) myeloma relative to others. Boxplots show the median and quartiles with the whiskers extending to the most extreme data point within 1.5 times the interquartile range.

Figure S7

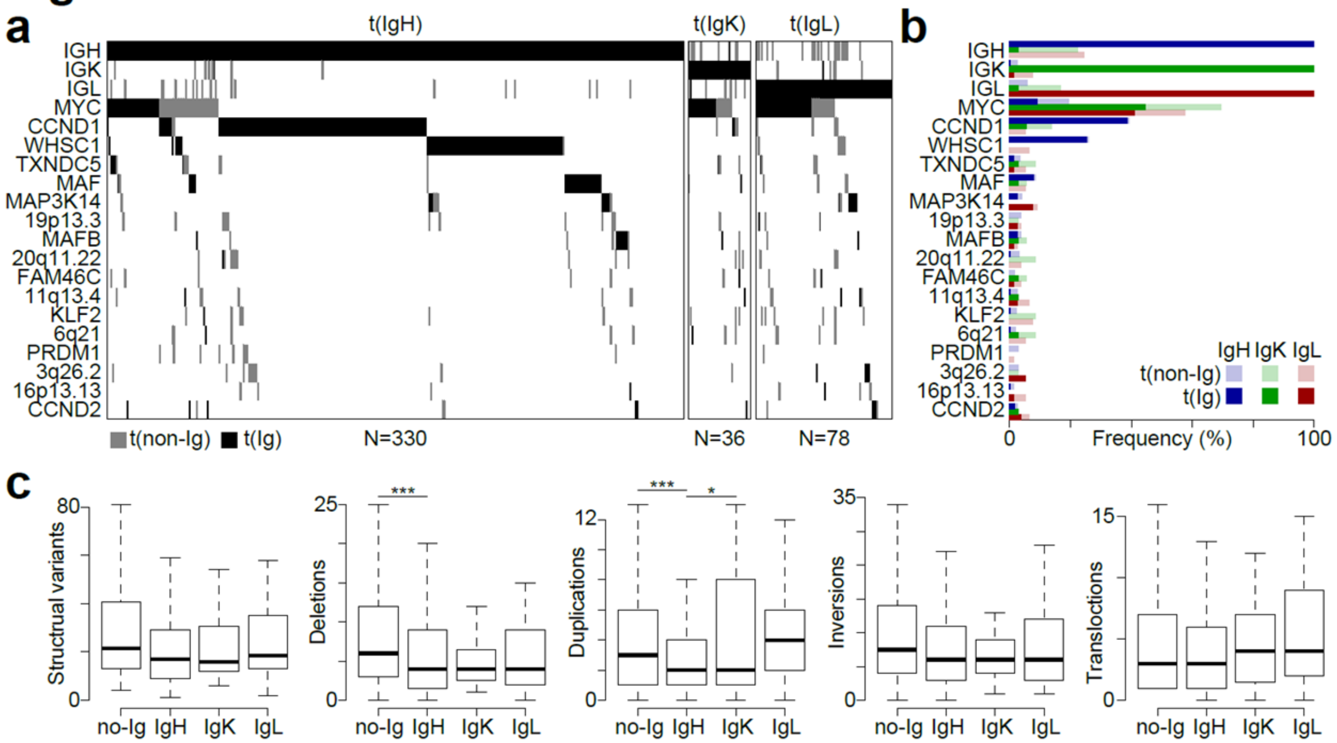

Figure S7. Structural variants in myeloma with immunoglobulin (Ig) translocations. **a** Waterfall plot of IgH (left), IgK (middle), and IgL (right) translocated myeloma with other translocations present in ≥2% of the newly diagnosed population. Both translocations directly to the respective Ig locus [t(Ig); black] and those that co-occur and are not translocated to the Ig locus [t(non-Ig); gray] are shown. **b** The frequency of IgH (blue), IgK (green) and IgL (red) translocations are shown for loci as in (**a**). t(Ig) translocations are shown in opaque colors and t(non-Ig) translocations are shown in translucent colors (see key bottom right). **c** Number of total structural variants (left), deletions (mid left), duplications (middle), inversions (mid right), and translocations (right) in myeloma with no-Ig (N=288), IgH (N=304), IgK (N=23), or IgL (N=54) translocations. Only myelomas with at least one translocation are shown and those with multiple Ig translocations are removed. **P* <0.05, ***P* <0.01, ****P* <0.001 analysis of variance with Tukey's post-hoc test. Boxplots show the median and quartiles with the whiskers extending to the most extreme data point within 1.5 times the interquartile range.

Figure S8

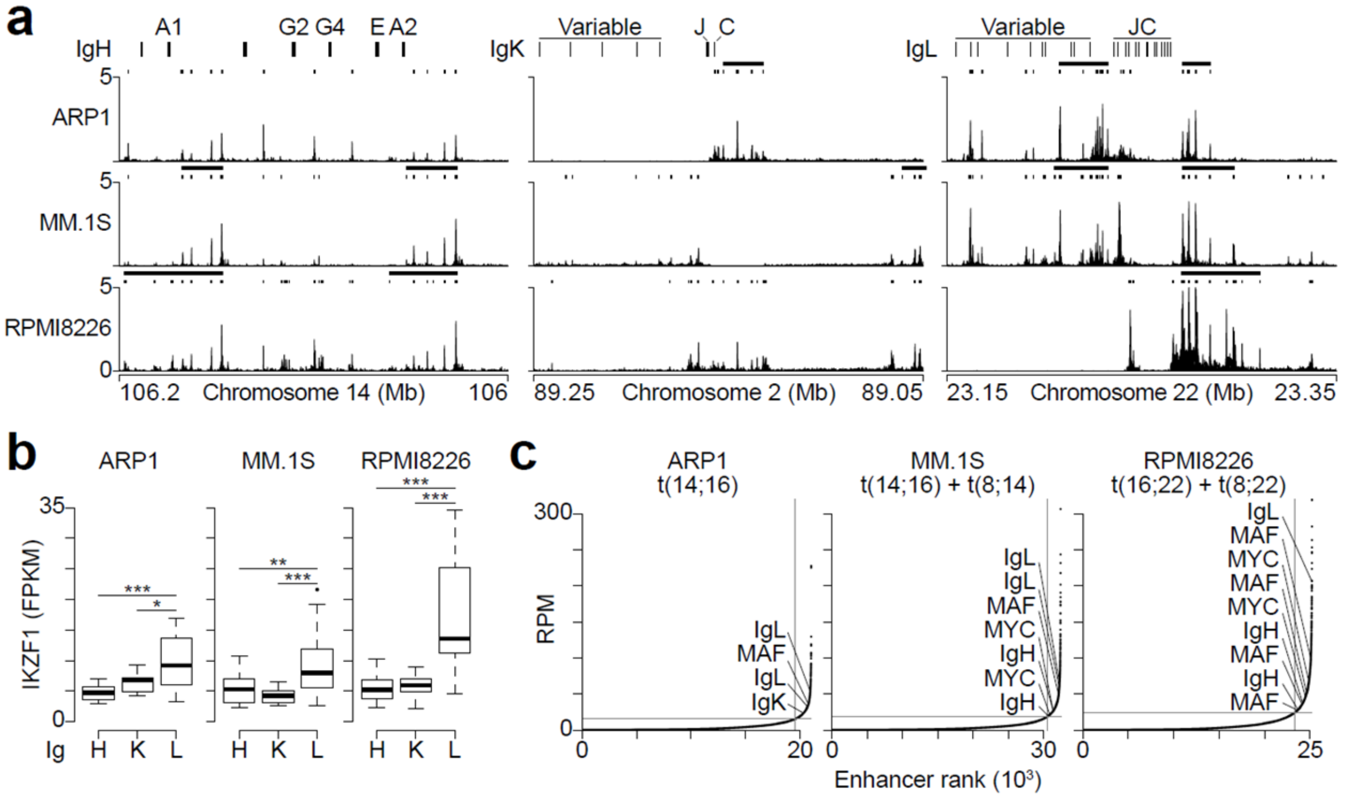

Figure S8. IKZF1 binds the IgL locus at some of the highest levels of the myeloma epigenome. **a** ChIP-seq of IKZF1 at the IgH (left), IgK (middle), and IgL (right) 3' loci for the myeloma cell lines ARP1 (top; IgK-expressing), MM.1S (middle; IgL-expressing), and RPMI8226 (bottom; IgL-expressing and IgL-translocated). Regions enriched for IKZF1 and high-occupancy clustered regions are denoted above each track and IKZF1 binding is shown on a common scale measured in reads per million. Immunoglobulin genes are shown (top) with variable, joining (J), and constant (C) regions labelled as well as the IgH constant regions. **b** IKZF1 binding at IgH (H), IgK (K), and IgL (L) regions shown in part **a** measured in fragments per kilobase per million (FPKM). Boxplots show the median and quartiles with the whiskers extending to the most extreme data point within 1.5 times the interquartile range. **c** Ranked order of IKZF1 bound regions in ARP1 (left), MM.1S (middle), and RPMI8226 (right) cells with high-occupancy clustered regions labelled for immunoglobulin and translocated loci. Data are shown on the GRCh37 genome.

Figure S9

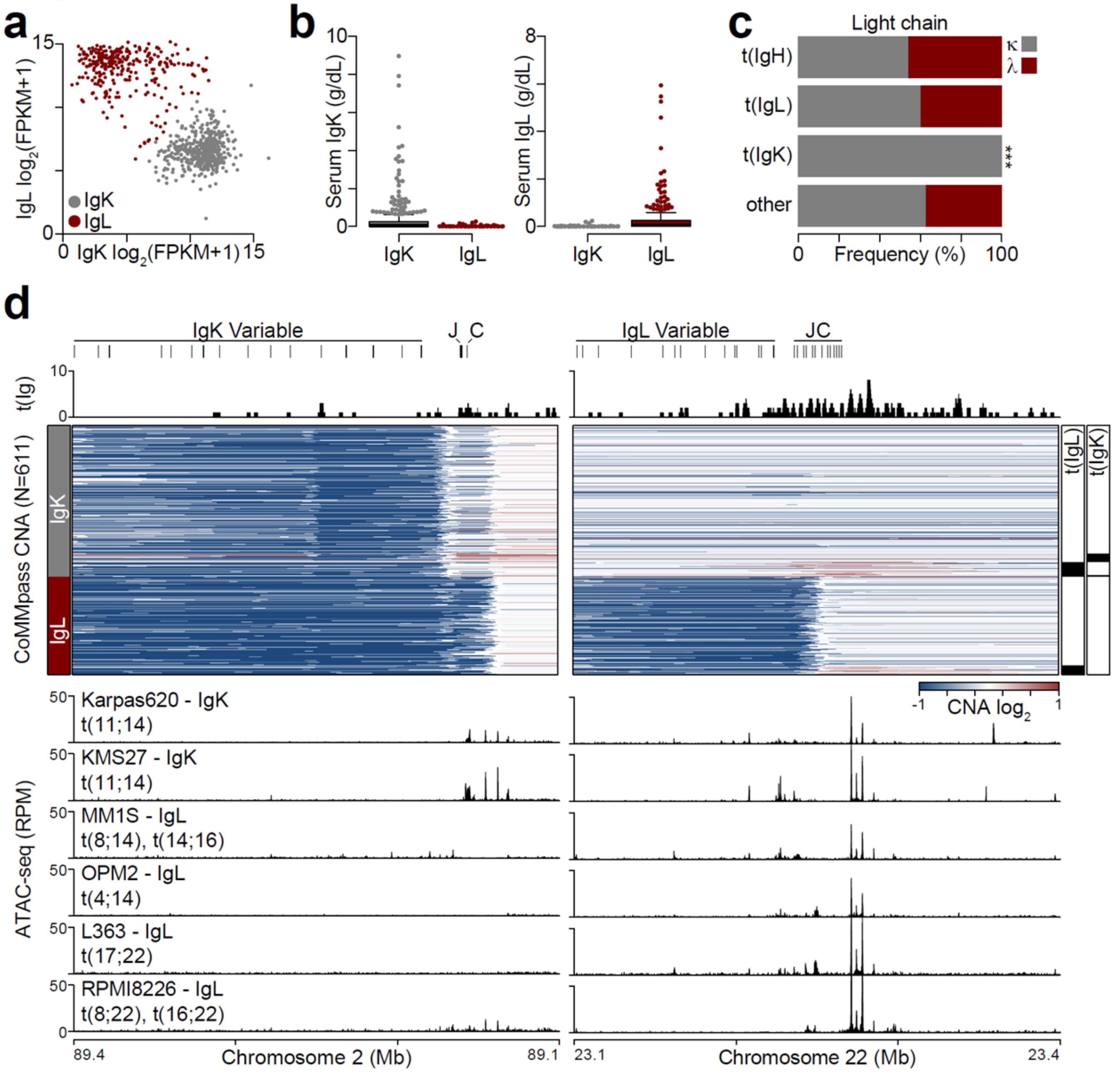

Figure S9. Deletion of the IgK 3’ enhancer in IgL expressing myeloma. **a** Plot of immunoglobulin kappa (IgK; gray) and immunoglobulin lambda (IgL; red) expression in 611 newly diagnosed myelomas with whole-genome and RNA-seq data. Color denotes the light chain expressed at the highest level. **b** Serum IgK (left) and IgL (right) for patients that express either IgK or IgL. **c** Frequency of IgK and IgL expression stratified by immunoglobulin translocation. **d** Genome plot of IgK and IgL loci showing translocations (t(Ig); top), copy number alterations (CNA) derived from long-insert whole genome sequencing for IgK-expressing (gray) and IgL-expressing (burgundy) myeloma (middle), and ATAC-seq in IgK and IgL expressing myeloma cell lines (bottom). ***P <0.001; Fisher’s exact test.
