# Supplementary material for: Multiple myeloma immunoglobulin lambda translocations portend poor prognosis
Source: Nat Commun. 2019 Apr 23;10:1911. doi: 10.1038/s41467-019-09555-6 (PMC6478743; doi:10.1038/s41467-019-09555-6)
Supplement: Supplementary file 2 — Description of Additional Supplementary Files [file 41467_2019_9555_MOESM2_ESM.docx]

**Description of Additional Supplementary Files**

File Name: Supplementary Data 1

Description: Somatic structural variants.

# sample: MMRF sample identifier

GRCH37.chr: start chromosome of structural variant (GRCh37) GRCH37.pos: start genomic position of structural variant (GRCh37) REF: DELLY: Reference Base

CIEND: DELLY: PE confidence interval around END CIPOS: DELLY: PE confidence interval around POS

CHR2: DELLY: end chromosome for structural variant (GRCh37) ENDPOSSV: DELLY: End position of the structural variant (GRCh37) PE: DELLY: Paired-end support of the structural variant

MAPQ: DELLY: Median mapping quality of paired-ends SR: DELLY: Split-read support

SRQ: DELLY: Split-read consensus alignment quality CONSENSUS: DELLY: Split-read consensus sequence CE: DELLY: Consensus sequence entropy

TR1.span: reads spanning the SV start site that indicate the cognate sequence TR1.reads: reads identifying the structural variant at the start site

TR2.span: reads spanning the SV end site that indicate the cognate sequence TR2.reads: reads identifying the structural variant at the start site

TR1.vaf: variant allele frequency for the SV start site TR2.vaf: variant allele frequency for the SV end site vaf: average variant allele frequency of the SV

File Name: Supplementary Data 2

Description: Genes differentially expressed in each expression subtype.

File Name: Supplementary Data 3

Description: Gene set enrichment analysis of expression subtype specific genes.

File Name: Supplementary Data 4

Description: Genes differentially expressed in t(IgL) myeloma.

File Name: Supplementary Data 5

Description: Gene set enrichment analysis of t(IgL) myeloma.

File Name: Supplementary Data 6

Description: Areas of sequencing anomalies removed from analysis
