# Supplementary material for: Multiple myeloma immunoglobulin lambda translocations portend poor prognosis
Source: Nat Commun. 2019 Apr 23;10:1911. doi: 10.1038/s41467-019-09555-6 (PMC6478743; doi:10.1038/s41467-019-09555-6)
Supplement: Supplementary file 9 — Reporting Summary [file 41467_2019_9555_MOESM9_ESM.pdf]

nature research

Corresponding author(s): PM Vertino and LH Boise

Reporting Summary

Nature Research wishes to improve the reproducibility of the work that we publish. This form provides structure for consistency and transparency in reporting. For further information on Nature Research policies, see Authors & Referees and the Editorial Policy Checklist.

Statistical parameters

When statistical analyses are reported, confirm that the following items are present in the relevant location (e.g. figure legend, table legend, main text, or Methods section).

n/a	Confirmed	
☐	☒	The exact sample size (n) for each experimental group/condition, given as a discrete number and unit of measurement
☐	☒	An indication of whether measurements were taken from distinct samples or whether the same sample was measured repeatedly
☐	☒	The statistical test(s) used AND whether they are one- or two-sided<br>*Only common tests should be described solely by name; describe more complex techniques in the Methods section.*
☐	☒	A description of all covariates tested
☐	☒	A description of any assumptions or corrections, such as tests of normality and adjustment for multiple comparisons
☐	☒	A full description of the statistics including central tendency (e.g. means) or other basic estimates (e.g. regression coefficient) AND variation (e.g. standard deviation) or associated estimates of uncertainty (e.g. confidence intervals)
☐	☒	For null hypothesis testing, the test statistic (e.g. F, t, r) with confidence intervals, effect sizes, degrees of freedom and P value noted<br>*Give P values as exact values whenever suitable.*
☒	☐	For Bayesian analysis, information on the choice of priors and Markov chain Monte Carlo settings
☐	☒	For hierarchical and complex designs, identification of the appropriate level for tests and full reporting of outcomes
☐	☒	Estimates of effect sizes (e.g. Cohen's d, Pearson's r), indicating how they were calculated
☐	☒	Clearly defined error bars<br>*State explicitly what error bars represent (e.g. SD, SE, CI)*

Our web collection on statistics for biologists may be useful.

Software and code

Policy information about availability of computer code

Data collection	Software used for processing the data contained within the manuscript are primarily open source and fully described (including version) in the methods
Data analysis	All software used for data analysis is open source and fully described in the methods (including version). All custom code is available upon request as stated in the methods.

For manuscripts utilizing custom algorithms or software that are central to the research but not yet described in published literature, software must be made available to editors/reviewers upon request. We strongly encourage code deposition in a community repository (e.g. GitHub). See the Nature Research guidelines for submitting code & software for further information.

Data

Policy information about availability of data

All manuscripts must include a data availability statement. This statement should provide the following information, where applicable:

- Accession codes, unique identifiers, or web links for publicly available datasets
- A list of figures that have associated raw data
- A description of any restrictions on data availability

CoMMpass data is deposited in dbGaP (phs000748.v6.p4) and summarized data can be accessed at https://research.themmrf.org/. ATAC-seq data is available under GEO GSE121912.

Field-specific reporting

Please select the best fit for your research. If you are not sure, read the appropriate sections before making your selection.

☒ Life sciences ☐ Behavioural & social sciences ☐ Ecological, evolutionary & environmental sciences

For a reference copy of the document with all sections, see nature.com/authors/policies/ReportingSummary-flat.pdf

Life sciences study design

All studies must disclose on these points even when the disclosure is negative.

Sample size	The CoMMpass study size was determined as 1,000 newly diagnosed patients prior to study initiation based on power analyses to identify structural variants, mutations, and gene expression subtypes of myeloma and their prognostic significance
Data exclusions	Copy number data from exome and/or whole genome sequencing data were eliminated for samples that did not pass copy number quality control
Replication	Replicate assays on select samples were performed to ensure exome, whole genome, and RNA-seq data was reproducible. Samples are collected from North American and European collection sites.
Randomization	Patient's were therapeutic regimens were determined by the treating physician. Inclusion criteria required patients be treated with either a proteasome inhibitor and/or an IMiD containing regimen. All samples were analyzed according to available material such that if more than 750,000 bone marrow CD138+ plasma cells were available then both RNA (RNA-seq) and DNA (exome-seq then whole genome sequencing) were extracted.
Blinding	Patients were de-identified and samples processing was thus blinded.

Reporting for specific materials, systems and methods

Materials & experimental systems

n/a	Involved in the study	
☒	☐	Unique biological materials
☐	☒	Antibodies
☐	☒	Eukaryotic cell lines
☒	☐	Palaeontology
☒	☐	Animals and other organisms
☐	☒	Human research participants

Methods

n/a	Involved in the study	
☐	☒	ChIP-seq
☒	☐	Flow cytometry
☒	☐	MRI-based neuroimaging

Antibodies

Antibodies used	anti-IKZF1 (GeneTex, GTX129438)
Validation	anti-IKZF1 validation data are described at http://www.genetex.com/Ikaros-antibody-GTX129438.html. This antibody has previously been published: https://www.ncbi.nlm.nih.gov/pubmed/26990993

Eukaryotic cell lines

Policy information about cell lines

Cell line source(s)	Cells were purchased from the American Type Culture Collection, the Japanese Collection of Research Bioresources, or Deutsche Sammlung von Mikroogransmen und Zellkulturen, except for ARP-1, which was obtained from the source (ref 60).
Authentication	Cell lines were genetically authenticated by the source.
Mycoplasma contamination	All cell lines tested negative for mycoplasma
Commonly misidentified lines (See ICLAC register)	*Name any commonly misidentified cell lines used in the study and provide a rationale for their use.*

Human research participants

Policy information about studies involving human research participants

Population characteristics	CoMMpass patients were all newly diagnosed with multiple myeloma and were recruited from North American and European collection sites. Patients were treated with physician's choice of regimens but had to include either a 1) proteasome inhibitor or 2) IMiD based regimen. Further details can be found in the manuscript (see Figure S2).
Recruitment	Patients were recruited by treating physicians through the MMRC.

ChIP-seq

Data deposition

☒ Confirm that both raw and final processed data have been deposited in a public database such as GEO.

☒ Confirm that you have deposited or provided access to graph files (e.g. BED files) for the called peaks.

Data access links *May remain private before publication.*	Go to https://www.ncbi.nlm.nih.gov/geo/query/acc.cgi?acc=GSE121912<br>Enter token yncrgyisbnarjwh into the box
Files in database submission	Raw FASTQ files and union of all peaks and normalized counts in each read
Genome browser session (e.g. UCSC)	http://www.broadinstitute.org/igv/projects/current/igv.php?sessionURL=http://aws-website-boiselab-sgghu.s3-website-us-east-1.amazonaws.com/MC-ATAC/ATAC.AWS.Len.ctrl.xml

Methodology

Replicates	Cell lines were analyzed once.
Sequencing depth	20-40 million paired-end reads
Antibodies	anti-IKZF1 (GeneTex, GTX129438).
Peak calling parameters	macs2 callpeak -g hs -q 0.01
Data quality	Fraction of reads in peaks was used as a quality statistic and this was routinely ranged from 10-20%
Software	All open source software was used to process and analyze the data as described in the methods. Custom pipeline and code is available upon request.
